# Longitudinal Associations of High‐Volume and Vigorous‐Intensity Exercise With Hip Fracture Risk in Men

**DOI:** 10.1002/jbmr.4624

**Published:** 2022-07-06

**Authors:** Marko T. Korhonen, Urho M. Kujala, Jyrki Kettunen, Olga V. Korhonen, Jaakko Kaprio, Seppo Sarna, Timo Törmäkangas

**Affiliations:** ^1^ Gerontology Research Center, Faculty of Sport and Health Sciences University of Jyväskylä Jyväskylä Finland; ^2^ Faculty of Sport and Health Sciences University of Jyväskylä Jyväskylä Finland; ^3^ Arcada University of Applied Sciences Helsinki Finland; ^4^ Faculty of Medicine and Health Technology Tampere University Tampere Finland; ^5^ Department of Public Health & Institute for Molecular Medicine FIMM University of Helsinki Helsinki Finland; ^6^ Department of Public Health University of Helsinki Helsinki Finland

**Keywords:** EXERCISE, AGING, FRACTURE PREVENTION, LONGITUDINAL STUDIES, OSTEOPOROSIS

## Abstract

Maintenance of vigorous exercise habits from young to old age is considered protective against hip fractures, but data on fracture risk in lifelong vigorous exercisers are lacking. This longitudinal cohort study examined the hazard of hip fractures in 1844 male former athletes and 1216 population controls and in relation to exercise volume and intensity in later years. Incident hip fractures after age 50 years were identified from hospital discharge register from 1972 to 2015. Exercise and covariate information was obtained from questionnaires administered in 1985, 1995, 2001, and 2008. Analyses were conducted using extended proportional hazards regression model for time‐dependent exposures and effects. During the mean ± SD follow‐up of 21.6 ± 10.3 years, 62 (3.4%) athletes and 38 (3.1%) controls sustained a hip fracture. Adjusted hazard ratio (HR) indicated no statistically significant difference between athletes and controls (0.84; 95% confidence interval [CI], 0.55–1.29). In subgroup analyses, adjusted HRs for athletes with recent high (≥15 metabolic equivalent hours [MET‐h]/week) and low (<15 MET‐h/week) exercise volume were 0.83 (95% CI, 0.46–1.48) and 1.04 (95% CI, 0.57–1.87), respectively, compared with controls. The adjusted HR was not statistically significant between athletes with low‐intensity exercise (<6 METs) and controls (1.08; 95% CI, 0.62–1.85). Athletes engaging in vigorous‐intensity exercise (≥6 METs at least 75 minutes/week) had initially 77% lower hazard rate (adjusted HR 0.23; 95% CI, 0.06–0.86) than controls. However, the HR was time‐dependent (adjusted HR 1.04; 95% CI, 1.01–1.07); by age 75 years the HRs for the athletes with vigorous‐intensity exercise reached the level of the controls, but after 85 years the HRs for these athletes increased approximately 1.3‐fold annually relative to the controls. In conclusion, these data suggest that continuation of vigorous‐intensity exercise is associated with lower HR of hip fracture up to old age. © 2022 The Authors. *Journal of Bone and Mineral Research* published by Wiley Periodicals LLC on behalf of American Society for Bone and Mineral Research (ASBMR).

## Introduction

Several population studies in older people indicate that a higher level of physical exercise in the later years is associated with lower risk of hip fracture.^(^ ^1^, ^2^, ^3^, ^4^ ^)^ Some studies which have assessed exercise habits from younger to older ages suggest that engagement in strenuous exercise during the formative years from adolescence to young adulthood may also have a significant protective effect on the risk of hip fracture in later life.^(^ ^5^, ^6^ ^)^ Evidence suggests that youth is the optimal time for intense exercise to improve peak bone mass,^(^ ^7^, ^8^, ^9^ ^)^ as well as balance and muscular strength,^(^ ^10^, ^11^ ^)^ which are important synergistic predictors of future falls and fractures.^(^ ^12^ ^)^ However, most training‐induced physical adaptations are transitory,^(^ ^13^, ^14^ ^)^ which suggests that the continuation of exercise throughout adult life may be necessary to sustain the gains made in young ages. Although the research on physical exercise and hip fractures in the general population have included participants with differing exercise patterns, there appear to be no follow‐up studies of older individuals who have engaged in vigorous levels of activity throughout their lives. Lack of fracture data on lifelong exercisers is a notable gap in the literature given that habitual engagement in relatively vigorous exercise is widely recommended as a means of promoting general health and function, while minimizing the risk of skeletal frailty and fractures.^(^ ^15^, ^16^ ^)^

Former competitive athletes represent a unique research cohort for studying the association of prolonged early‐life training exposure with bone traits and fractures later in life.^(^ ^17^, ^18^, ^19^, ^20^, ^21^ ^)^ In an earlier examination of the present male Finnish former elite athlete cohort, the hazard of hip fracture was slightly lower in the former athletes than population controls aged 50 years or older.^(^ ^22^ ^)^ Other studies conducted in Sweden have also shown a trend toward lower hip fracture hazard in retired older male athletes compared to peers in the general population.^(^ ^23^, ^24^, ^25^ ^)^ However, these few available studies in former athletes may have lacked statistical power due to having no or a low number of participants over age 70–75 years; ie, a life phase when hip fracture incidence rates begin to increase exponentially.^(^ ^26^ ^)^ Moreover, the previous studies with former athletes have not addressed the possible influence on hip fracture risk of variation in exercise volume and intensity after an active sport career.

Consequently, this study continued and extended the follow‐up of the male Finnish former athlete cohort to provide additional information on the association of long‐term exercise habits with the risk of hip fractures in older ages. The objectives were as follows:

1. *Primary objective* was to investigate the association of hip fracture hazard with high‐volume and vigorous‐intensity exercise determined at four time points from midlife to old age.
2. *Secondary objective* was to examine whether the original findings on fracture risk between the entire group of athletes and their controls^(^ ^22^ ^)^ remain unchanged over an additional 10 years of observational time when most of the participants had reached an age when hip fractures become more common.
3. *The tertiary (supportive and exploratory) objective* was to evaluate the relation of the exercise volume and intensity to hip fractures in pooled cohort of athletes and controls.

Our hypothesis was that among former athletes, continuation of exercise at both high‐volume and vigorous intensity levels would be related to lower hazard for hip fracture compared with less active controls. We also expected that hazard for hip fracture would be lower in the entire group of athletes than in their controls.

## Subjects and Methods

### Study population

The selection of athletes and their sports events and population controls matched for age and area of residence has been detailed in previous publications.^(^ ^17^, ^27^, ^28^ ^)^ Briefly, the original athlete cohort consisted of 2448 former male athletes who had represented Finland at least once in the Olympic Games, World or European Championships, or inter‐country competitions between 1920 and 1965 in endurance (middle and long distance running, cross‐country skiing), power (shot put, discus throw, javelin, hammer throw, weight lifting, wrestling, boxing), team game (soccer, basketball, ice hockey), other track and field (jumping, sprinting, hurdling, decathlon), or shooting events. Shooters were not included in the present study. The athletes were identified through athletic organizations’ yearbooks and registers and 97.7% of eligible participants were traced. All the selected sports disciplines and training regimens included weight‐bearing dynamic activities, with probable load on the proximal femur. Due to the low number of fracture cases in the different sports categories, and hence to maximize statistical power, all the athletes were collapsed into one group for the main analyses.

The control group consisted originally of 1712 healthy Finnish men from the same age cohort and area of residence as the athletes. Controls were selected from among men classified as having A1 health (completely healthy) at the medical examination into military service at age 20 years. This allowed the analyses to be conducted free from possible confounding effect on the relationship between lifelong exercise and fracture incidence caused by including individuals with chronic diseases and impairments at a young age.^(^ ^29^ ^)^ Selection of the controls was carried out when 85.3% of the athletes were identified. No controls for athletes traced afterward were obtained that partially explains lower number of the controls than athletes in the present study.^(^ ^27^ ^)^ Participants were traced primarily through records of local parishes. All study participants were of European ancestry.

The present analyses were limited to participants aged 50 years and older, as only a few fragility fractures occur at younger ages.^(^ ^26^ ^)^ In this updated analysis, in contrast to the previous study by Kettunen and colleagues,^(^ ^22^ ^)^ we excluded an additional 303 athletes and 251 controls due to fracture or death before age 50 years or prior to the start of the fracture follow‐up in 1972, or due to missing birth and death data, or to relocation abroad during the follow‐up. After these exclusions, the final study population for the first fracture analyses comprised 1844 athletes and 1216 controls. To control for the potential effect of lifetime work history (eg, occupational physical loading), we collected data on the participants’ longest held occupation mainly from the Central Population Registry of Finland and also from questionnaires. For the occupation‐based analyses, we categorized the participants into six groups: executives, clerical staff, skilled workers, unskilled workers, farmers, and other or unspecified occupation.^(^ ^30^ ^)^ The study was approved by the Ethics Committee of the University of Jyväskylä and conformed to the principles of the Declaration of Helsinki. Permission for record linkages was received from the Finnish Institute for Health and Welfare and Statistics Finland.

### Questionnaire data

Our new analysis included a subgroup of athletes and controls who participated in later follow‐up questionnaire studies conducted in 1985 (*n* = 1985), 1995 (*n* = 1431), 2001 (*n* = 1136), and 2008 (*n* = 575). Overall, 67% of the participants responded at least once over the four waves of surveys. The questionnaires included items on personal characteristics known to affect fracture risk, including anthropometry (height, weight), living situation (married/cohabiting, living alone, in 1985 and 1995), alcohol use (g/month), smoking status (current, former, never), and physical exercise. Leisure‐time physical exercise was assessed by items on the average intensity, average duration, and monthly frequency of exercise activities during the previous year.^(^ ^31^, ^32^ ^)^ Average exercise intensity and total exercise volume were expressed as metabolic equivalent units (METs: work metabolic rate/resting metabolic rate) and by a sum score of MET hours of exercise per week (MET‐h/week), respectively.^(^ ^33^, ^34^ ^)^ For the subgroup analyses, physical exercise was divided into two exercise volume and two exercise intensity categories. The cut‐point for high exercise volume was defined as at least 15 MET‐h/week, an exercise level that has been found to associate with increased bone mineral density (BMD) at the proximal femur in adult men (corresponds to at least twice the minimum recommended level of physical activity^(^ ^35^ ^)^). The cut‐point for vigorous exercise intensity was defined as MET score of 6 or greater.^(^ ^36^, ^37^, ^38^ ^)^ In addition, the time criterion for vigorous exercise intensity was set to at least 75 minutes per week (if lower, the participant was placed in the low‐intensity group).^(^ ^37^ ^)^ Occupational and non‐sportive activities (eg, snow clearing, mowing the lawn, wood cutting, cleaning) were not included in the measure.

### Identification of hip fractures

The follow‐up data for hip fractures were drawn from the Care Register for Health (including all hospitals in Finland) and the search covered the records of inpatient care from January 1, 1972 to December 31, 2015. The hospital discharge records were linked with information on dates of deaths collected from the National Death Register, Statistics Finland. Hip fractures were identified by the International Classification of Diseases (ICD) codes (International Classification of Diseases, Revision 8 [ICD‐8] for years 1972–1986, code 820; International Classification of Diseases, Ninth Revision [ICD‐9] for 1987–1995, code 820; International Classification of Diseases and Related Health Problems, 10th Revision [ICD‐10] for 1996–2015, codes S72.0–S72.2). Only fragility hip fractures caused by moderate to minimal trauma (eg, any fall from a standing height or less) were included in this study. The literature reports that over 90% of all hip fractures are caused by falls from standing height.^(^ ^39^ ^)^ Fractures caused by transport and vehicular accidents or other major traumas were identified and excluded using ICD injury codes (ICD‐8 codes E807–E846, E916–E928; ICD‐9 codes E800A–E830A, E920A–E928A; ICD‐10 codes V01–V99, W10–W19). Participants with the high‐energy hip fractures were excluded from the study. Fractures were collected from the register based on both primary and secondary fracture diagnoses. If a participant had sustained multiple hip fractures (two participants), only the first fracture was included in the analyses. The Care Register for Health has been acknowledged as a very reliable source in its accuracy and completeness for the diagnosis of severe injuries such as hip fractures.^(^ ^40^, ^41^, ^42^, ^43^ ^)^

### Statistical analyses

As sample characteristics we report means and proportions together with 95% confidence intervals (CIs). As descriptive statistics of fractures data, we report person‐years of exposure, number of fractures, and fracture rates per 100 person‐years. We used incidence rate ratios (IRRs) together with their 95% CIs to examine an unadjusted relationship across pairs of groups with the control group as the reference group. We analyzed group differences in average ages at fracture with variance analysis.

IRRs provide a crude estimate for the development of fractures because they do not account for differences in timing of the fractures. Thus, to study the importance of participants’ age at the fracture hospitalization, we used the Cox regression model (see the Methodological Supplement for more details). As a secondary objective, we studied hip fracture hazard between all former athletes and controls using Cox proportional hazards regression models adjusted for occupation. This analysis included participants with fracture data available for the years 1972 to 2015.

In our primary objective analysis, we used Cox regression models to evaluate the association of exercise variables and covariates on hip fractures, including data available from follow‐up questionnaires in 1985, 1995, 2001, and 2008. Comparisons were made between athletes in two categories of exercise volume (<15 MET‐h/week versus ≥15 MET‐h/week) and exercise intensity (<6 METs versus ≥6 METs at least 75 minutes/week) and controls. We used age as survival time, and the participants were followed starting from the first questionnaire administration (athletes) or from 1985 (controls) until time of death, diagnosis of hip fracture, or end of follow‐up on December 31, 2015. The exercise categories were allowed to vary as time‐dependent exposures,^(^ ^44^ ^)^ and we also modeled the association of the exercise with the ratio of fracture hazards as a time‐dependent effect based on a polynomial function of time.^(^ ^45^ ^)^

We report the proportional hazards model results as hazard ratios (HRs), which are pairwise comparisons of hazard rates (*h*) for different study groups. For a time‐dependent effect, the hazard ratio of the covariate is separated into two components: (i) an effect that remains constant over time, and (ii) an interaction effect of the covariate with a function of time. The former defines the level of the covariate effect at the point where the follow‐up time is zero, whereas the latter captures the changes occurring in the HR over time. More details are given in the Methodological Supplement. If, based on the scaled Schoenfeld residual test and plot,^(^ ^45^ ^)^ significant HR modification was observed, we reported the time‐dependent effects. The models were adjusted for height, weight, living situation, occupational class, alcohol use, and smoking status. All the covariates, excluding occupational class, were treated as time‐dependent exposures. Assessment of nonresponse using missing data indicators suggested that missingness was not related to any of the variables under study, and hence missing data in confounders were not imputed. For the study objectives, Nelson‐Aalen cumulative hazard plots were used to illustrate the unadjusted accumulation number of expected fractures in the study groups over time.

In the supplementary and exploratory analysis, the same multiple regression model was also used to compare fracture hazard rates of the two exercise volume and two exercise intensity groups created from the pooled population of athletes and controls. All the analyses were limited to those aged 90 years or below to avoid problems due to sparse data at high age. Statistical significance level was set to 0.05. Descriptive analyses were performed using SPSS Statistics software (v. 26.0; IBM Corp, Armonk, NY, USA). Fracture‐time analyses were done using a custom script utilizing the packages survival (version 3.1‐12),^(^ ^46^ ^)^ lubridate (version 1.7.9),^(^ ^47^ ^)^ epiR (version 1.0‐15)^(^ ^48^ ^)^ and emmeans (version 1.5.1)^(^ ^49^ ^)^ in the R programming environment (version 4.0.2; R Foundation for Statistical Computing, Vienna, Austria; https://www.r-project.org/).^(^ ^50^ ^)^ More details related to the data and methodology can be found in the Methodological Supplement.

## Results

Background characteristics for all available participants in the first questionnaire study in 1985 are shown in Table 1. The former athletes were slightly older, taller, and smoked less than controls, whereas body weight, body mass index (BMI), living situation, and alcohol use did not differ between the groups. More former athletes than controls were in the executive and clerical occupational classes. As reported earlier,^(^ ^22^ ^)^ the former athletes had a twofold greater exercise volume (MET‐h/week) and higher exercise intensity (MET), duration and frequency than controls (all *p* < 0.001). Significant differences in exercise volume and intensity as well as participation rate in the high volume (≥15 MET‐h/week) and vigorous intensity of exercise (≥6 METs at least 75 minutes/week) in favor of the former athletes persisted at all time points (Fig. S1).

**Table 1:** Characteristics of the Study Participants in the First Questionnaire Study in 1985

Characteristic	Former athletes	Controls
Age (years), mean (95% CI)	56.9 (56.3–57.5)	55.1 (54.4–55.9)
Height (cm), mean (95% CI)	176.4 (176.0–176.8)	174.9 (174.5–175.3)
Weight (kg), mean (95% CI)	81.4 (80.7, 82.1)	80.9 (80.0, 81.8)
BMI (kg/m^2^), mean (95% CI)	26.1 (25.9–26.3)	26.4 (26.1–26.6)
Living situation, % (95% CI)		
Married/cohabitating	84.7 (82.8–86.8)	82.7 (80.0–85.4)
Living alone	15.3 (13.2–17.2)	17.3 (14.6–20.0)
Occupational class, % (95% CI)		
Executives	24.2 (21.8–26.6)	11.5 (9.2–13.7)
Clerical staff	41.4 (38.6–44.1)	26.0 (22.9–29.1)
Skilled workers	28.5 (26.0–31.0)	41.8 (38.3–45.2)
Unskilled workers	2.0 (1.2–2.8)	5.1 (3.5–6.6)
Farmers	3.9 (2.9–5.0)	15.6 (13.0–18.2)
Other	0.0 (0.0–0.0)	0.1 (0.0–0.4)
Alcohol use (g/month), mean (95% CI)	426 (394–459)	398 (354–441)
Smoking status, % (95% CI)		
Current	20.9 (18.7–23.3)	30.7 (27.3–34.0)
Former	29.2 (26.6–31.7)	40.8 (37.4–44.4)
Never	49.9 (47.0–52.7)	28.5 (25.2–31.7)
Leisure‐time exercise, mean (95% CI)		
Exercise duration/week (hours)	4.0 (3.8–4.3)	2.7 (2.5–3.0)
Frequency/week, *n*	3.4 (3.3–3.6)	2.7 (2.5–2.9)
Total exercise volume, MET‐h/week^a^	30.0 (28.0–32.0)	15.0 (13.3–16.7)
Average exercise intensity, MET^b^	7.1 (6.9–7.3)	5.3 (5.1–5.4)

BMI = body mass index; MET = metabolic equivalent.

^a^ Total exercise volume (MET‐h/week) was calculated from the product of average exercise intensity × duration × frequency.

^b^ Average exercise intensity is reported in MET units (ie, energy demands of habitual exercise level as multiples of resting metabolic rate). One MET equals approximately 3.5 mL of oxygen uptake per kilogram of body weight per minute, or to energy expenditure of 1 kcal per kilogram of body weight per hour.

Descriptive statistics of fractures by different study groups are shown in Table 2. The present updated analyses are based on 40,986 and 25, 146 person‐years of exposure for athletes and controls, respectively. The average follow‐up period for the entire cohort was 21.6 years (quartiles: 13.7, 29.7). During the follow‐up, 62 (3.4%) former athletes and 38 (3.1%) controls sustained a hip fracture. The fracture incidence rate ratio (IRR), which indicates the fracture rate throughout the follow‐up period without considering the time‐dependent effect, was identical between all athletes and controls. Moreover, the IRR estimates between the athlete subgroups (high and low exercise volume and intensity) and controls were statistically nonsignificant.

**Table 2:** Descriptive Statistics of Fractures in Different Study Groups Between January 1, 1972, and December 31, 2015

Group (number of participants)	Person‐years of exposure	Number of fractures	Fracture rate (95% CI)^a^	IRR (95% CI)
Total (*n* = 3060)	66,132	100	0.15 (0.12–0.18)	–
Athlete versus control				
Ctrl (*n* = 1216)	25,146	38	0.15 (0.11–0.21)	ref
Athlete (*n* = 1844)	40,986	62	0.15 (0.12–0.19)	1.00 (0.66–1.54)
Exercise volume (cut‐point: 15 MET‐h/week)^b^ ^,^ ^c^				
Ctrl (*n* = 783)	13,592	26	0.19 (0.12–0.28)	ref
High‐volume athlete (*n* = 746)	13,702	22	0.16 (0.10–0.24)	0.84 (0.45–1.54)
Low‐volume athlete (*n* = 559)	9592	25	0.26 (0.17–0.38)	1.36 (0.75–2.46)
Exercise intensity (cut‐point: 6 METs ≥75 minutes/week)^c^ ^,^ ^d^				
Ctrl (*n* = 783)	13,592	26	0.19 (0.12–0.28)	ref
Vigorous‐intensity athlete (*n* = 630)	10,692	12	0.11 (0.06–0.20)	0.59 (0.27–1.20)
Low‐intensity athlete (*n* = 679)	12,639	35	0.28 (0.19–0.39)	1.45 (0.85–2.50)

CI = confidence interval; IRR = incidence rate ratio; ref, reference category (IRR is 1).

^a^ Fracture rate per 100 person‐years.

^b^ Total exercise volume (metabolic equivalent [MET]‐h/week) was calculated from the product of average exercise intensity × duration × frequency. Exercise volume and intensity data were gathered from questionnaires administered in 1985, 1995, 2001, and 2008.

^c^ Participants with missing data between 1985 and 2008 (*n* = 970), person‐years: 11,999, number of fractures: 27, and fracture rate: 0.23 (95% CI, 0.15–0.33).

^d^ Average exercise intensity is reported in MET units (i.e., energy demands of habitual exercise level as multiples of resting metabolic rate).

**Fig. 1:**
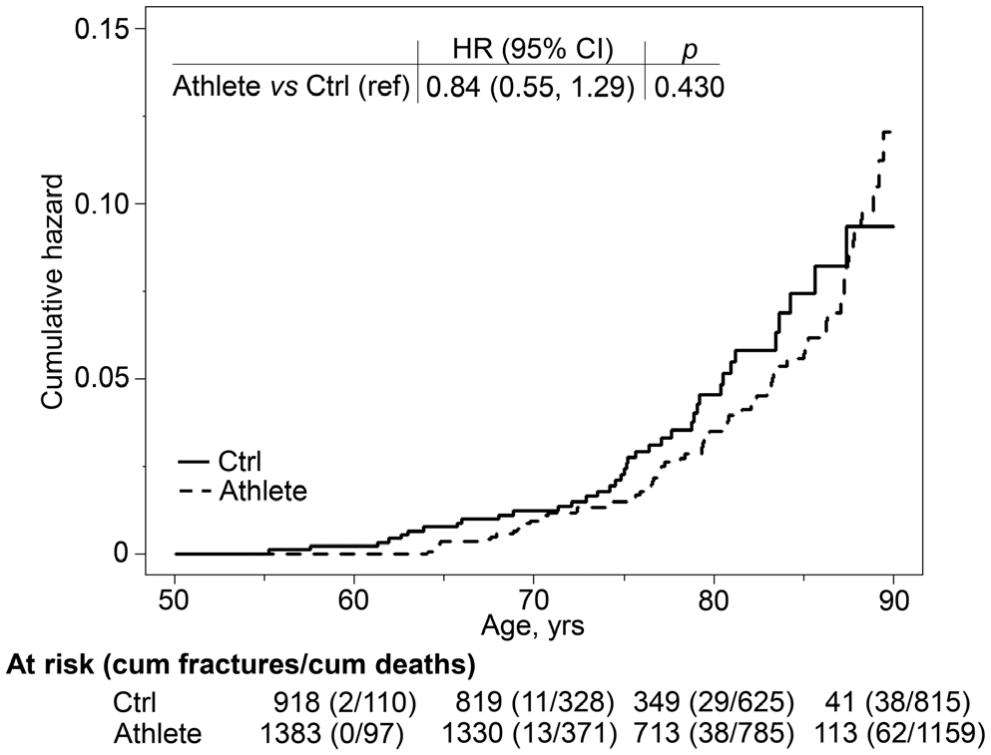
Nelson‐Aalen cumulative fracture hazard curves of hip fractures for controls (Ctrl) and all former athletes (Athlete). HR adjusted for occupation and its 95% CI are displayed at the top. Hip fractures were followed from January 1, 1972 until time of death, diagnosis of hip fracture, or end of follow‐up on December 31, 2015. HR = hazard ratio; CI = confidence interval.

Figure 1 shows the unadjusted cumulative fracture incidence curves for athletes and controls in our secondary objective analysis. The HR adjusted for occupation indicated no statistically significant difference in relative fracture hazard rates between athletes and controls (*p* = 0.430). However, mean age at the time of the fracture event was 77.6 years (95% CI, 75.8–79.5) among the athletes compared to 73.8 years (95% CI, 71.2–76.4) among the controls. The difference of 3.84 years (95% CI, 0.65–7.04) was statistically significant (*p* = 0.019).

**Fig. 2:**
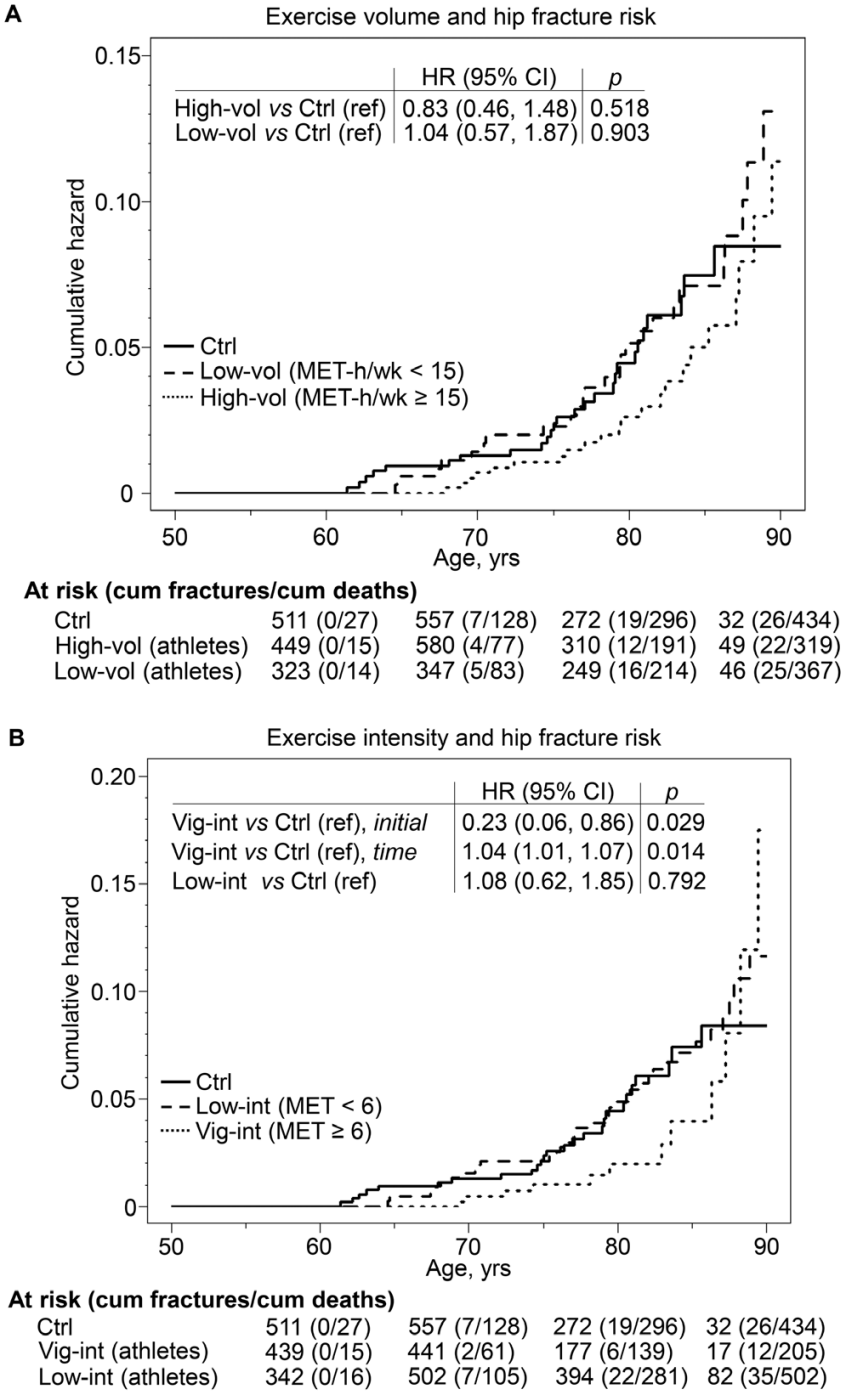
Nelson‐Aalen cumulative fracture hazard curves of hip fractures for controls (Ctrl), and athletes divided into two subgroups according to total exercise volume (*A*) and average exercise intensity (*B*). HRs and their 95% CIs are shown in the inset tables. The models were controlled for body height, body weight, living situation, occupational class, alcohol use, and smoking status. Nelson‐Aalen curves of hip fracture risks for former athletes were adjusted by possible changes in exercise level during follow‐up. Time‐dependent effect observed for exercise intensity (*B*) is partitioned into two components: initial HR is the hazard ratio at the start of the follow‐up period and time HR refers to risk modification over time. Hip fractures were followed starting from the first questionnaire participation (athletes) or from 1985 (controls) until time of death, diagnosis of hip fracture, or end of follow‐up on December 31, 2015. Exercise and other characteristics were obtained from questionnaire studies in 1985, 1995, 2001, and 2008. See Table 1 footnote for description of calculation of exercise volume and intensity with MET values. HR = hazard ratio; CI = confidence interval; MET = metabolic equivalent.

Cumulative hip fracture incidence curves for the time‐dependent measurements of total exercise volume exposure in our primary objective analysis are shown in Fig. 2*A*. The multiple covariate‐adjusted fracture hazard rate for the former athletes with exercise volume of at least 15 MET‐h/week and for those with less than 15 MET‐h/week was not significantly different in comparison to controls. In addition, mean age at fracture occurrence in the athletes with higher (78.8 years; 95% CI, 75.8–81.9) and lower (77.5 years; 95% CI, 74.7–80.4) exercise volume was delayed on average by 3 to 4 years (point estimate), from that in controls (74.6 years; 95% CI, 71.8–77.4; *p* = 0.046 and 0.154, respectively).

The association of the two time‐dependent exercise intensity levels with hip fracture incidence in our primary objective analysis is shown in Fig. 2*B*. Covariate‐adjusted fracture hazard rate for the former athletes who maintained vigorous exercise intensity of ≥6 METs for at least 75 minutes/week was initially 77% lower than for controls (HR, 0.23; 95% CI, 0.06–0.86). However, the association of vigorous‐intensity exercise with fracture risk showed time‐dependency toward old age. Relative to controls, the hazard rate of fracture was lower for the vigorously active athletes until about age 75 years, after which the hazard rates remained similar up to about age 85 years. The athletes’ rate rose heavily in a curvilinear fashion with an annual 1.3‐fold hazard rate increase so that the cumulative hazard was about the same as in the other two groups by the age 90 years. At age 85 years, 21.3% of vigorous‐intensity athletes and 14.7% of controls were still at risk for fractures. The hazard rate of fracture among athletes who did lower intensity exercise, ie, <6 METs, was not significantly different from that of the controls or vigorously exercising athlete group. Hip fractures occurred among the vigorous exercise intensity athletes on average 5.5 years later (80.1 years; 95% CI, 76.0–84.2) and among the lower exercise intensity athletes 2.8 years later (77.5 years; 95% CI, 75.1–79.9) than controls (74.6 years; 95% CI, 71.8–77.4); the difference was statistically significant between the vigorously active athletes and controls (*p* = 0.031), but not between the low‐intensity athletes and controls (*p* = 0.128, respectively). A sensitivity analyses where the athletes and controls were treated identically with regard to start of being at risk did not materially change the results of exercise volume and intensity in primary hypothesis study (see Methodological Supplement).

For our tertiary objective analysis, we combined the cohort of athletes with the controls and formed two groups based on exercise volume and intensity to examine the association with covariate‐adjusted fracture hazard ratios (Fig. S2). In this analysis, we did not find evidence supporting a lower fracture hazard in the high than in low exercise volume group (see Fig. S2 *A* for effect size). Moreover, no statistically significant difference was observed in the level effect of the fracture hazard rate in the vigorous compared to low exercise intensity group (HR, 0.68; 95% CI, 0.37–1.26). However, a significant time‐dependent curvilinear increase was found in the fracture hazard rate in the vigorous compared to low exercise intensity group: using the approximate Wald test (see Methodological Supplement), a protective effect in favor of the vigorously active participants was observed until approximately 67 years of age. Between ages 67 and 84 years the hazard rates did not differ significantly; however, from the age of 84 years onward the hazard rate rose in a curvilinear fashion in the vigorous relative to lower exercise intensity group (hazard rate increased approximately 1.4‐fold annually), similar to the result we found in the primary objective analysis (Fig. 2*B*).

## Discussion

This extended follow‐up of the Finnish male former elite athlete cohort examined, to our knowledge for the first time, whether early‐life athletic participation and continued physical exercise at higher volume and intensity levels in later life are associated with hip fracture risk. In line with the original study of this same cohort,^(^ ^22^ ^)^ the relative risk of fracture in our secondary objective analysis was not significantly different in the combined athlete group from the controls. A novel finding of the study was that among the athletes, the maintenance of vigorous‐intensity exercise was associated with a 77% reduction in the initial hazard rate for hip fractures compared with controls, whereas higher exercise volume was not associated with a significant reduction in fracture hazard rate in our primary objective analyses. However, the upswing in fracture hazard rate for the oldest athletes (≥85 years) in the vigorous exercise intensity group, although based on a small sample, suggests that high age may modify the association between vigorous exercise and fracture hazard rate.

Evidence from some studies,^(^ ^22^, ^23^, ^51^, ^52^ ^)^ but not all studies,^(^ ^53^, ^54^ ^)^ in former high‐performance athletes suggests that years of training and competing in weight‐bearing sports can lead to positive adaptations in femur bone mass, size, and structure that are not completely lost even many decades after cessation of training. However, the answer to the critical question whether former athleticism can ultimately reduce the risk of hip fractures in old age remains unclear. The original fracture follow‐up of the present cohort as well as earlier reports from Sweden have shown either no difference in hip fracture risk in retired male athletes compared to matched population controls or only a nonsignificant trend toward a lower risk in the athletes.^(^ ^23^, ^24^, ^54^ ^)^. Here the additional 10 years of follow‐up data allowed us to follow the entire cohort into a more advanced age during which the proportion of hip fractures increased by 54% percent. The present result was in line with the previous finding in this cohort,^(^ ^22^ ^)^ which found the hip fracture hazard rate was slightly, but not significantly, lower in the combined athlete group than controls.

Important new (primary and tertiary) objectives of this study were to examine whether variation in total exercise volume and average exercise intensity in later life is associated with hip fracture hazard. Our hypothesis that fracture hazard would be associated with both higher volume and more intense exercise was only partially supported. The results indicated that among the athletes, a higher exercise volume of at least 15 MET‐h/week was not statistically significantly associated with lower fracture hazard rate compared to controls. In turn, the athletes who engaged in vigorous intensity exercise ≥6 METs at least 75 minutes/week initially had a 77% lower hip fracture hazard rate than controls. However, this hazard changed around age 75 years, when the fracture hazard rate became similar to that of the control group. After age 85 years, the rate of the vigorously active athletes, which until then had remained lower, began to rise. Thus, among the vigorous‐intensity athletes, the cumulative fracture hazard remained notably lower than among controls even up to the ninth decade (see Fig. 2*B*).

Very few studies have dealt with the importance of the volume and intensity of exercise as determinants of the fracture risk. However, a recent study found a significant 35% lower hazard rate of hip fractures among vigorously active older men and women (aged 65–90 years) participating in long‐distance cross‐country ski races as compared to age‐matched controls.^(^ ^55^ ^)^ Our findings also showed consistency with a prospective 21‐year study of a Finnish male population reporting that participation in vigorous exercise at baseline (age 50 years) was associated with a 62% reduction in the hazard rate of hip fracture. In that investigation exercise volume and fracture hazard rate were not associated, except that among the men in the second quartile of exercise volume, the rate was reduced.^(^ ^38^ ^)^ A meta‐analysis of 13 prospective population studies concluded that moderate‐to‐vigorous exercise is related to a hip fracture risk reduction of 45% and 38% among men and women.^(^ ^3^ ^)^ More recently several studies,^(^ ^1^, ^2^, ^4^ ^)^ but not all studies,^(^ ^36^ ^)^ have confirmed the protective effect of exercise during later adulthood against hip fractures in men. Note, however, that many population studies have classified vigorous exercise according to overall volume (including household and/or occupational tasks) rather than intensity of leisure physical activity. It must also be emphasized that the majority of population studies have not examined time‐varying effects of exercise on fracture hazard and lack data on participation in sportive activities in young ages and may thus not be directly comparable with our results.

Our study allows no conclusions to be drawn on the mechanisms by which the maintenance of relatively vigorous exercise intensity might be more protective against later hip fractures than high total exercise volume. However, studies in animals have indicated that bone adaptation to exercise seems to be intensity‐dependent, whereas the duration and number of loading cycles is of less importance.^(^ ^56^, ^57^ ^)^ In addition, some studies in middle‐aged and older men indicate that the maintenance of a high osteogenic index or loading intensity, rather than total time spent in physical activities, is associated with greater skeletal benefits at the loaded sites.^(^ ^58^, ^59^, ^60^ ^)^ Therefore, it is possible that among former athletes, even a modest amount of participation in vigorous‐intensity exercise (75 minutes/week), may have been effective in counteracting age‐related decline in femoral neck strength, thus reducing the probability of a hip fracture in the case of a fall. Another explanation might be that continued vigorous exercise helps to protect against fractures through other mechanisms such as preserved rapid muscle force capacity^(^ ^20^ ^)^ and dynamic postural balance^(^ ^61^ ^)^ that could reduce the frequency of falls and/or facilitate effective protective responses during a fall.^(^ ^12^ ^)^ Furthermore, the association may also reflect better functional and health status,^(^ ^27^ ^)^ which could contribute to maintaining intensive activity and thus influence the fracture risk.

On the other hand, our model of the time‐dependent effects of exposure suggests that the advantage with respect to fracture hazard of vigorous exercise in athletes may decrease after approximately age 85 years and the cumulative hazard rate increase to the level of the controls. It should be remembered that over half of all hip fractures occur in individuals who are not osteoporotic but are classified as having osteopenia or low bone mass.^(^ ^62^ ^)^ Thus, it may be that in very old age some former athletes reach the fracture threshold at the same time as they confront increasing physiological limitations due to aging processes (eg, hormonal changes), whereas their wish to engage in year‐round leisure exercise exposes them to hazardous situations, such as slippery winter conditions, where falls and fractures can more easily occur.^(^ ^63^, ^64^ ^)^

A possible confounding factor when comparing former athletes and population‐based controls is that the nonathletic group includes some participants with vigorous leisure activity (≥6 METs). To address this issue, we performed the tertiary objective analysis to evaluate fracture hazard in the two exercise volume and intensity groups in the pooled cohort of athletes and controls. With this reclassification the effect from higher exercise intensity on fracture HR appeared to partly support the finding from our primary hypothesis study: the time effect indicated hazard modification in the vigorous‐intensity versus low‐intensity groups in the early and final follow‐up years. The finding that the results of time effect remained similar as in the primary objective analysis is somewhat surprising and unclear given that the controls most probably had trained at a lower intensity than the athletes in young ages. Therefore, it could be speculated that although training in weight‐bearing sports in youth is important in achieving high starting bone strength and physical abilities, maintenance of vigorous‐intensity activity in later years may also play a significant role in hip fracture protection in non‐athletes.

Studying former elite athletes, who in later life were still exercising intensively relative to age‐appropriate maximum capability, provided us with a “natural experimental setting” that would be difficult to emulate with any other approaches to study of lifetime vigorous exercise. Key methodological strengths in this study include the long follow‐up time, a complete national cohort of international‐level former athletes in the main sporting events, an age‐ and area‐matched general population control group, and reliable national hospital register data on fractures and trauma types (low versus high energy). A further important strength of our study was the evaluation of exercise and covariates up to four time points during follow‐up that allowed us to capture potential variation in exercise exposure or confounding factors from mid‐to‐late life; fracture‐preventive association would have remained undetected had we not considered the time‐dependent effect of exercise on fracture hazard rates.

The main limitation of this study is the relatively small number of fracture events, which may explain some of the null results and prevented us from carrying out subgroup analyses on athletes subjected to different loading modalities and multiple doses including extreme training to clarify ideal and safe upper limit of exercise in old ages. One limitation is the use of self‐reports of physical exercise, which are susceptible to errors.^(^ ^65^ ^)^ We also acknowledge that MET and MET‐h/week values in the absence of information on the exercise type must be considered at best as crude proxy measures of bone loading intensity and volume. Furthermore, the questionnaire studies did not collect data on the age at which competitive sporting activities were commenced and ceased. However, in a recent follow‐up survey of this cohort, the athletes (*n* = 214) reported having begun regular training at 10.9 ± 3.6 years of age and competing at 13.5 ± 3.4 years of age (unpublished data), which is in line with other studies of former athletes.^(^ ^52^ ^)^ The mean age when ceasing to compete was 38.9 ± 15.6 years, with some athletes continuing their sports at a competition level as veterans. A further limitation of the study is that our database of fractures did not include information about the circumstances causing the injury, and possible comorbid conditions and medications which could affect the ability to exercise and change the risk of falls and fractures. However, the response rates in different time points were only slightly lower in controls than former athletes. Our analysis of missing data found no major differences in terms of age or socioeconomic variables, suggesting that the missing data was random subset of data. Finally, although our conclusions can be criticized for being limited to the most highly trained and best adapted male athletes, it should also be remembered that training regimens have evolved over the past decades. The former athletes in this study participated in competitions over half a century ago and it is possible that the training level during their active careers was lower than it is among modern professional athletes and perhaps more comparable to that of committed recreational exercisers.

## Conclusion

In conclusion, no difference was observed in fracture hazard rates between the entire male athlete cohort and controls. Among a subgroup of the former athletes, the maintenance of vigorous exercise intensity was associated with reduced initial fracture hazard rate (77%), whereas total exercise volume had no significant association with hazard rate. Furthermore, in the vigorous‐intensity athletes the fracture events were postponed on average by 5.5 years compared with controls. Our analysis suggests, however, a further possible interaction between age and exercise for fracture hazard whereby the protective association on hazard with vigorous‐intensity exercise may be attenuated from approximately age 85 years onward. Together, these findings suggest that vigorous‐intensity exercise throughout life, not sports participation in youth alone, is associated with decreased hazard of hip fracture in later years. The finding that the fracture‐protective association of vigorous exercise diminished in very old age may imply that careful attention should be paid to safe exercise forms and conditions, but not that participation in sportive activities should be avoided.

## Author Contributions

**Marko T. Korhonen:** Funding acquisition; investigation; methodology; writing – original draft; writing – review and editing. **Urho M. Kujala:** Investigation; methodology; project administration; writing – original draft; writing – review and editing. **Jyrki Kettunen:** Investigation; methodology; writing – review and editing. **Olga V. Korhonen:** Investigation; writing – original draft; writing – review and editing. **Jaakko Kaprio:** Funding acquisition; investigation; methodology; project administration; writing – original draft; writing – review and editing. **Seppo Sarna:** Investigation; methodology; project administration; writing – original draft; writing – review and editing. **Timo Törmäkangas:** Funding acquisition; investigation; methodology; writing – original draft; writing – review and editing.

## Conflict of interest

All authors state that they have no conflicts of interest.

## Data availability

The data that support the findings of this study are available upon reasonable request from the corresponding author. The registry data are not provided outside the University of Jyväskylä or the University of Helsinki due to privacy and ethical restrictions.

### Peer Review

The peer review history for this article is available at https://publons.com/publon/10.1002/jbmr.4624.

## Supporting information


**Appendix S1.** Supplementary Information
Methodological Supplement

**Fig. S1.** Total exercise volume (*A*) and average exercise intensity (*B*) by group, and proportion of athletes and controls reaching cut points of high total exercise volume of ≥15 MET‐hour/week (*C*) and vigorous average exercise intensity of ≥6 METs at least 75 minutes/week (*D*) at different time points. Values within bars are means (*A, B*) and mean percentages (*C, D*) with 95% confidence intervals in parentheses. *p* < 0.001 in all pairwise comparisons between groups (*A–D*). Total exercise volume (*A*: cf. Kontro et al. Eur J Sport Sci, DOI: 10.1080/17461391.2020.1761889).

**Fig. S2.** Nelson‐Aalen cumulative fracture hazard curves of hip fractures for the pooled sample (athletes plus controls) stratified into two groups based on total exercise volume (*A*) and average exercise intensity (Panel B). Hazard ratios (HRs) and their 95% confidence intervals (CIs) are shown in the inset tables. The models were controlled for occupation, height, weight, living situation, alcohol use and smoking history. Nelson‐Aalen curves of hip fracture risks were adjusted by possible changes in exercise level during follow‐up. Time‐dependent effect observed for exercise intensity (*B*) is partitioned into two components: initial HR is the hazard ratio at the start of the follow‐up period and time HR refers to risk modification over time. Hip fractures were followed starting from the first questionnaire participation until time of death, diagnosis of hip fracture or end of follow‐up on December 31, 2015. Exercise and other characteristics were obtained from questionnaire studies in 1985, 1995, 2001 and 2008. See Table 1 footnote for description of calculation of exercise volume and intensity with MET (metabolic equivalent) values.
