# Supplementary material for: Longitudinal Associations of High‐Volume and Vigorous‐Intensity Exercise With Hip Fracture Risk in Men
Source: J Bone Miner Res. 2022 Jul 6;37(8):1562–70. doi: 10.1002/jbmr.4624 (PMC9544739; doi:10.1002/jbmr.4624)
Supplement: Supplementary file 1 — Appendix S1. Supplementary Information Methodological Supplement [file JBMR-37-1562-s001.pdf]

Methodological supplement

Longitudinal associations of high-volume and vigorous-intensity exercise with hip fracture risk in men

Timo Törmäkangas

Event time modelling

In the main analyses we examined the relationship between exercise volume and intensity covariates on event time outcomes using the extended proportional hazards regression model[1] (see Therneau and Grambsch 2000) that permits the use of both *time-dependent covariates* and *time-dependent effects*. The outcome in the model consists of two variables, the *event time* and *event status*. Because the model includes time-dependent covariates, it is convenient to reconstruct the data by using the counting process approach (Andersen & Gill 1982) to be described below. We define the event of interest, which is the same for all hypotheses, and then define how event time was constructed for the three types of hypotheses addressed in this study, and then consider in detail the model used in the analysis.

Event

The event of interest was defined as *fragility hip fracture* obtained from the Care Register for Health (including all hospitals in Finland). From the fracture diagnoses our focus was on for primary and secondary diagnoses of first hip fracture with the following-ICD codes: ICD-8 for the years 1972-1986, codes 820; ICD-9 for 1987-1995, codes 820; ICD-10 for 1996-2015, codes S72.0-S72.2. All data for participants with transportation accidents and other types of high-energy traumas causing hip fractures was excluded from analysis based on ICD injury codes. We also considered two events that prevented us from observing a fracture: *death* and *end of follow-up* (without sustaining a hip fracture) due to study end were considered as right censoring events[2].

Event time

Because of the large age range in the starting point of the fracture follow-up in 1972 (mean age in 1972: 47 years, std. deviation: 13 years), it was not likely that calendar time would have constituted a meaningful unit of time, and we chose to use the age (computed as time differences between dates and expressed as years) of the participants instead. Use of age permitted relating the participants against each other so that their biological contribution was placed on a uniformly interpretable scale. Event time has two important points: the beginning, origin, and termination. We next define termination of follow-up time because this definition was the same for all study hypotheses.

---

[1] The model is called extended because the original model formulation by Cox (1972) did not consider time-dependent covariates or covariate-time interaction effects.

[2] Having not sustained a fracture prior to Dec. 31st, 2015, was considered censored from the right, because a fracture might have occurred, had the follow-up time been extended. Death can be considered a competing risk because it terminates the follow-up time but is not a hip fracture. Since we were only interested in the association of exercise covariates on fracture hazard, we do not report hazard ratios for mortality, which is equivalent to treating death as a censoring event.

Event time termination

We considered three kinds of endpoint events terminating the follow-up for each participant. These events were the same for all study objects. The follow-up time was monitored over the period starting from Jan. 1st, 1972 and ending in Dec. 31st, 2015. If a participant had not sustained a hip fracture and was still alive prior to the end limit of the interval, he was categorized as censored. If the participant had died prior to that end of the follow-up window, he was categorized as dead. If a fracture was observed prior to the end of the follow-up window, he was categorized as having sustained a fracture and exited the follow-up (i.e., was no longer at risk for fracture). For the event of death, follow-up termination time was defined as the age from birth to date of death. For the event of fracture, termination time was the age from birth to recorded fracture date. For the participants surviving (censorings) until the end of follow-up, termination time was the age from birth to Dec. 31st, 2015. The flow of participant into the three objectives are shown in Figure MS1.

Event time origin, ending and structuring

Questionnaires of physical exercise and other characteristics were administered in four occasions in 1985, 1995, 2001 and 2008 (see Sarna et al. 1997). This data was used in the definition of event time origin.

Primary hypothesis study

The former male athlete vs. control group status was known for the participants since the initial group allocation, and hence controls remained in the control group throughout the study. For the primary hypothesis study event time origin for the control group participants was defined as the age from birth to the first questionnaire administration in 1985 if they participated in at least one measurement wave (not necessarily 1985). However, for younger participants the origin was delayed until the participant had reached the age of 50 years.

To account for the athletes' later life physical exercise level, we utilized information about their total exercise volume and average exercise intensity from the four questionnaire administration waves. The athlete exercise grouping was determined by using a variable-specific cut-point for low and high groups (exercise volume: ≥ 15 MET-h/week, exercise intensity: MET score ≥ 6 at least 75 min/wk), and it was permitted to change according to observed value in each measurement wave. An athlete's origin of event time was defined as the participant's age from birth to the earliest available measurement wave in which the participant took part and was aged at least 50 years. Using age as the origin of time in this way permitted accounting for truncation related to *delayed entry* into the follow-up. The approach is similar to that described in Kalbfleisch and Prentice (2002, section 1.6) and arises naturally in the counting process data format.

As an illustration, consider the theoretical[3] participants shown in Figure MS2. Dashed line is used to indicate time from birth to milestones of the study. Solid horizontal line refers to time spent in study follow-up. Participants A-E were in the athlete group and F-H in the control group. For athlete group participants A, B, C and E, the origin was age in the 1985 wave, and for participant D age in 1995 as D did not participate in the 1985 measurement. For the control group participants (F-H) the origin was the age at the 1985 measurement.

According to the counting process format the follow-up time was split into up to four intervals corresponding to waves of measurement for exercise volume and intensity[4]. Follow-up time for participant A consisted of

---

[3] The examples are illustrative but not actual cases in the data. Because of privacy concerns, we refrain from showing actual data.

[4] If a participant died or sustained a fracture before any measurement wave, there would be data for less than four intervals available.

four periods[5], where the exercise volume and intensity variables took the values observed at the beginning of each interval ($x_{A,85}$, $x_{A,95}$, $x_{A,01}$ and $x_{A,08}$)[6]. Thus, the participant would spend this interval at risk in corresponding group according to the value of the exercise variable. The intervals were constructed similarly for participant B, except that he was not in the 1995 measurement wave, and we used the value from 1985 measurement as a replacement for the 1995-2001 interval (thus, observed pattern: $x_{A,85}$, -, $x_{A,01}$, and $x_{A,08}$ was replaced with the pattern $x_{A,85}$, $x_{A,85}$, $x_{A,01}$, and $x_{A,08}$). Participant C was observed only from the 1985 measurement until death prior to the 1995 measurement. Note that, because the participant died, we did not attempt to fill in the later measurement for this participant. Participant D was observed for three intervals before study end, as the time origin was set at the 1995 measurement. Participant E was observed for two intervals prior to fracture between the 1995 and 2001 measurement waves. Note also that some participants may have missed the first measurement rounds if their age was below 50 years in 1985 and/or 1995 measurements (see Figure MS9).

We proceeded similarly with the participants in the control group, except that if they had missing data from the initial measurement waves, they were still counted as if they participated in the 1985 measurement because their exercise grouping was known from the study set up. For example, in Figure MS2 participants F and G participated from the 1995 measurement onwards, although the origin of follow-up time is still the 1985 wave. Table MS1 shows the frequencies related to various observation patterns across the measurement waves and the replacements. As can be seen from the table, a large part of the data was either intact or missing a single observation. Figure MS5 illustrates how a conventional data matrix is brought into the counting process format. Figures MS6-MS8 of this supplement show the lexis diagrams[7] linking the calendar time with the age of the participants over the follow-up time.

Secondary hypothesis study

Follow-up time construction for the secondary hypothesis was simpler, as the athlete-control status of the participants was known from initial group allocation. Entry to follow-up was determined by age at the start of the monitoring interval (Jan. 1st, 1972). If by that date the participant was aged 50 years or older[8], his follow-up period began from that date. If the participant was younger than 50 years, we waited until the date of the 50th birthday to start the follow-up. The 50-year limit was used because previous research suggests that among men fracture risk begins to rise approximately from that age onwards (e.g., Hernlund et al. 2013). However, in our data we noted that first observed fractures were sustained at approximately age 60 years, indicating that the choice was useful, because no known previous fractures that we were able to determine from fracture records were excluded from analysis.

Figure MS3 shows the observational data for the same cases as above for the secondary objective. The age of participants B, C, F, and H was 50 years, and participants A and F older than 50 years when the fracture registry started in 1972, so their entry age was 50 years or greater, while participants E and G were younger than 50 years, and their follow-up origin began later during the follow-up when their age reach the 50-year

---

[5] For participant A the periods span from (a_{85}, a_{95}], (a_{95}, a_{01}], (a_{01}, a_{08}] and (a_{08}, $a_{Fracture}$], where a_x is the age in wave x interval endpoint. Curved opening bracket (parenthesis) is read as starting from but not including the value shown. Squared closing bracket is read as ending in and including the value shown. For continuous data this convention is used to prevent counting the same individual twice in the period intersections.

[6] We use the symbol X to in place of the exercise volume or intensity variables.

[7] When more than one timescale is important in the same study, the lexis diagram can be used to show the relationship between the scales. For further information see e.g., section 6.4 in Clayton & Hills 1994.

[8] We considered it rare for fragility fractures to occur at younger ages, we set the approximate minimum age for start of follow-up at 50 years. Generally, fragility fractures have been found to begin to accumulate at around 50-60 years of age (Hernlund et al. 2013).

limit. For the secondary hypothesis study the participants remained in either the control or athlete group throughout the follow-up period. Thus, in this analysis the grouping covariate was not time dependent.

Tertiary hypothesis study

Follow-up construction proceeded similarly to the setting for primary hypothesis. The only difference was that the exercise variables were derived from the actual exercise volume and intensity of the participants in both the control and athlete groups. In other words, the sample was redistributed (pooled) into low and high volume and low and vigorous intensity categories irrespective of the original athlete or control status. The illustrative Figure MS4 shows that time arrangement was the same for participants A-E in the athlete group. For participants in the control group, we now account for their presence from the earliest wave observations from the exercise data became available. For example, participants F and G first participated in the 1995 measurement, and their event time origin was based on this measurement, while participant H participated first in the 1985 wave, which was his time origin. For all three cases total follow-up time was broken into three periods, and exercise variable values were updated according to the value obtained from the respective questionnaire administration at the start of each period.

Bias considerations

The study design is a longitudinal cohort study. Several sources of bias have been identified for cohort studies. For example, all types of fracture detection could be influenced by *health care access bias*. As hip fracture is a serious adversity requiring surgery, it seems impossible to avoid hospital admission. We expect there to be no or at most minimal *detection bias*, and as the annual coverage of the Care Register for Health has been found excellent (98 %) for hip fractures (Sund, 2012), and we expect no or at most a minimal number of fractures missing from the records. Possibly some minor injuries were operated without surgical care in private clinics, and these may have not been included in the fracture register. We also expect there to be minimal *time-lag bias*, as the time that the participants spent at risk for fracture from the origin to termination of the follow-up time has been carefully tailored for the three objectives of our study. Finally, *immortal time bias* refers to potential bias when measurements of variable *Z* made in the future (i.e., after origin of the follow-up time) are used to define groups under study. Then, participants are said to spend immortal time in the follow-up until the measurement of *Z*. Thus, to correct for the problem the origin for the follow-up must be defined so that the covariates are also measured at the origin. There are various ways to analytically deal with this form of bias (see e.g., Zheng et al 2005), but it is best if the origin of time can be defined so that no immortal time is included in the observation times. We defined the follow-up time origins specifically for each objective of the study as shown above, and in this manner attempted to avoid the problem of immortal time, because follow-up times reflect the true time spent at risk for fracture from the actual time point when the covariates were first measured.

For example, for the primary hypothesis, exercise volume and intensity were first measured by questionnaire in 1985 and we started the follow-up clock from the age of the first observed questionnaire participation (1985, 1995, 2001, 2008) for the athletes. As the control status was known from before, follow-up for all control group participants started from their age in 1985. This could potentially introduce immortal time bias if the control participants spent more time under observation. We conducted a sensitivity analysis by harmonizing inclusion of the control group participants into analysis based on their first observed exercise measurement time point (1985-2008); i.e., entry into follow-up (at-risk for fracture) was based on the same criterion as for the athletes. For exercise volume we observed the following hazard ratios: $HR_{\text{High-vol/Ctrl}}$ = 0.82 (95 % CI: 0.46, 1.46), p = 0.501; $HR_{\text{Low-vol/Ctrl}}$ = 1.05 (95 % CI: 0.59, 1.89), p = 0.860. For exercise intensity, the

vigorous-intensity athlete-control group comparison hazard ratios were: HR_{level} = 0.24 (95 % CI: 0.68, 0.84, p = 0.026) and HR_{time} = 1.04 (95 % CI: 1.01, 1.06, p = 0.017) and for the low-intensity athlete-control group comparison: *HR* = 1.03 (95 % CI: 0.61, 1.84, p = 0.832). So, the results of the manuscript seem to represent a slightly conservative estimate of the associations[9].

We examined *selection bias* through participation rates, which we found to be relatively similar across those who responded to the questionnaire in the athlete and control groups, and in terms of equal representation with respect to age and occupational grouping. Table MS2 shows the number and percentage of participants responding to the exercise items of the questionnaire in each measurement wave among the participants surviving up to each measurement without having sustained a hip fracture. The percentages indicate that while all control group participants took part in the primary objective analysis, a lower portion of athletes was available. However, based on examination of age (Figure MS13 and MS14) and occupational grouping (Table MS3) in the athlete group, we found no substantial evidence of bias in between favouring those who were observed over those who had missing data. As the control group was smaller than the athlete group, this meant that the groups sizes were closer to each other for the event time analysis.

Figures MS13 and MS14 show density plots of the participant age measured in 1972. The age distributions, although not exactly alike[10], appear similar among those who responded to the exercise variables compared to those who had missing values. This suggests that in terms of age the participants were adequately represented in the four time points of the study. Table MS3 shows the frequencies for the occupational categories cross-tabulated against measurement years within those with observed and missing MET-h/wk value. The table indicates that among the athletes who participated in questionnaire studies, executives, clericals and skilled workers categories had higher response frequencies while unskilled workers and farmer occupations were more frequently selected among the controls. Most importantly, the occupational class frequencies in 1972, i.e., when there were no missing data, were similar to those observed in the subsequent questionnaire administrations. Also, within each questionnaire administration the relative frequencies (percentages) among athletes and controls were similar between those with missing and observed values.

Data format and the proportional hazards model

We structured the data using the counting process data format (see e.g., section 3.7 in Therneau and Grambsch 2000). This format recognizes that event-times have an origin and an ending and that for each participant more than one non-overlapping period can be included in the analysis. This is useful for likelihood construction when incorporating time-dependent covariates in analysis, and when accounting for left truncation (delayed entry). We next consider the link between covariates and hazard within a period for a single participant. Within period, covariates are related to hazard via the hazard function defined[11] as:

$$\lambda(t_i) = \lambda_0(t) exp\left(\mathbf{u}_{i(t)}^{\mathrm{T}} \boldsymbol{\beta}_{(t)}\right), \quad i = 1, \ldots, N, \tag{1}$$

where *N* is the number of participants, *t* indexes the age, $\lambda_0(t)$ is the baseline hazard function, u_i is a *p*-element vector of fixed observed covariate values characterizing participant *i* at time *t*, and **β** is the corresponding *p*-

[9] The probability related to information loss using uncorrected vs. corrected control-group entry-time is proportional to 1.9×10^{-12}. Although the uncorrected model has superior fit, the impact of the hazard ratio estimates was minor.
[10] We considered representativeness was poorest in the 2008 measurement, when the numbers of available MET-h/wk measurements were the lowest of those eligible for analysis at the time point (Table MS2), as the participants who had missing data were on average older than the ones with observed data.
[11] For presenting quantities in the equations: letter in italics refers to a scalar (e.g., *z*), letter in bold typeface refers to vector (e.g., *z*), and Greek letters refer to coefficients (e.g., β).

element vector of regression coefficients. In equation 1, the terms in $exp(\mathbf{u}_{i(t)}^{\mathrm{T}}\boldsymbol{\beta}_{(t)})$ include the hazard ratios (HR) of the covariates. Since we focus on binary indicator variables, we consider interpretation only in that case[12]. For a two-category indicator, the HR characterizes the ratio of hazard rates, *h*(*t*), in the two groups being compared at time *t*. In the latter case we can interpret the HR directly or make an inference about the group-specific hazard rates. For example, if at *t* the hazard rate in the group of interest is $h_1 = \frac{1}{30}$ and the rate in the reference group is $h_0 = \frac{2}{30}$, we can calculate the hazard ratio as $HR = \frac{h_1}{h_0} = \frac{1/30}{2/30} = \frac{1}{2}$. We can also say that the hazard rate in the group of interest was 50 % of that of the reference group. The corresponding model regression coefficient estimate is approximately $\hat{\beta} = -0.69$, which yields the save hazard ratio upon exponentiation.

In the analyses we utilized three types of models. The *time-fixed variables and effects* model (M_1) corresponded to the conventional proportional hazards model, where covariate values were determined at the start of the study, and they remain unaltered throughout the follow-up time. Also, a single, time-averaged effect represented the covariate over follow-up time. The predictor component of the model in equation (1) was written as:

$$\mathbf{u}_i(t)^{\mathrm{T}}\boldsymbol{\beta}(t) = \mathbf{x}_i{}^{\mathrm{T}}\boldsymbol{\beta}_{\mathbf{x}} + \boldsymbol{z}_i{}^{\mathrm{T}}\boldsymbol{\beta}_{\mathbf{z}}, \qquad (2)$$

where x_i is an s-element vector of observed values for covariates of interest for participant *i* over the complete follow-up time, z_i an *r*-element vector of adjusting covariates, $\boldsymbol{\beta}$ were vectors of regression coefficients quantifying the natural logarithm of the hazard ratio for the two covariate vectors, respectively.

The second model type (M_2) was based on *time-dependent covariates and time-fixed effects*. The model effects of the linear predictor included the time-dependent fixed effects:

$$\mathbf{u}_i(t)^{\mathrm{T}}\boldsymbol{\beta}(t) = \mathbf{x}_i(t)^{\mathrm{T}}\boldsymbol{\beta}_{\mathbf{x}} + \boldsymbol{z}_i(t)^{\mathrm{T}}\boldsymbol{\beta}_{\mathbf{z}}, \qquad (3)$$

where $x_i(t)$ is an s-element vector of observed values for covariates of interest for participant *i* at time *t*, $z_i(t)$ an *r*-element vector of adjusting covariates at time *t*. Note that although the covariates are permitted to change over time, the regression coefficients remain constant over the full follow-up time.

The third model utilized both *time-dependent covariates and effects* (M_3), written as:

$$\mathbf{u}_i(t)^{\mathrm{T}}\boldsymbol{\beta}(t) = \mathbf{x}_i(t)^{\mathrm{T}}\boldsymbol{\beta}_{\mathbf{x}} + \boldsymbol{z}_i(t)^{\mathrm{T}}\boldsymbol{\beta}_{\mathbf{z}} + \mathbf{w}_i(t)^{\mathrm{T}}\boldsymbol{\beta}_{\mathrm{T}}g(t), \qquad (4)$$

where the first two terms are as for the model of equation (3), and the last term is an interaction term including at least one covariate from the covariate vector $x_i(t)$ of the observed time-dependent covariate values of interest at time *t* now in a *q*-element (where[13] $1 \leq q \leq s$) vector w_i multiplied by a pre-specified function *g* of time *t*, and $\boldsymbol{\beta}_{\mathrm{T}}$ is the *q*-element vector of regression coefficients. Note that the last term on the right-hand side of equation (4) is not a traditional interaction term[14] because in the hazards model it is used as an ordering component in the likelihood construction over ordered time.

---

[12] For a continuous variable the HR quantifies the covariate association for a unit-increase in the covariate value.
[13] If $q = 0$, model of equation (4) simplifies to model of equation (3).
[14] By traditional interaction term we mean e.g., an interaction variable computed for linear regression model, which can be computed prior to analysis as the product of two independent variables. For proper accommodation of a time-dependent effect in analysis, computation is performed at run-time during the analysis (for example, in the R programming environment the function *coxph* utilizes a time-transformation option for time-interactions of covariates).

For the models the continuous exercise volume and intensity variables were coded into two indicator variables[15] for each exercise quality as:

$$1_{low}(\text{volume}) \coloneqq \begin{cases} 1 & \text{MET}-\text{h/wk} < 15 \\ 0 & \text{MET}-\text{h/wk} \geq 15 \\ 0 & \text{Control,} \end{cases}$$

(5)

$$1_{high}(\text{volume}) \coloneqq \begin{cases} 0 & \text{MET}-\text{h/wk} < 15 \\ 1 & \text{MET}-\text{h/wk} \geq 15 \\ 0 & \text{Control,} \end{cases}$$

and

$$1_{low}(\text{intensity}) \coloneqq \begin{cases} 1 & \text{MET score} < 6 \\ 0 & \text{MET score} \geq 6 \\ 0 & \text{Control} \end{cases}$$

(6)

$$1_{vigorous}(\text{intensity}) \coloneqq \begin{cases} 0 & \text{MET score} < 6 \\ 1 & \text{MET score} \geq 6 \\ 0 & \text{Control.} \end{cases}$$

For the analyses of the primary objective, model M_2 was used for exercise volume and model M_3 for exercise intensity. We can write the model M_2 part involving the exercise volume indicators as:

$$\mathbf{x}_i{}^{\mathrm{T}}\boldsymbol{\beta}_{\mathbf{x}} = 1_{low}(\text{volume})_i\beta_{\text{low volume}} + 1_{high}(\text{volume})_i\beta_{\text{high volume}} \tag{7}$$

and model M_3 part involving exercise intensity as:

$$\mathbf{x}_i{}^{\mathrm{T}}\boldsymbol{\beta}_{\mathbf{x}} = 1_{low}(\text{intensity})_i\beta_{\text{low}-\text{intensity}} + 1_{vigorous}(\text{intensity})_i\beta_{\text{vigorous}-\text{intensity}},$$

$$\mathbf{w}_i(t)^{\mathrm{T}}g(t)\boldsymbol{\beta}_{\mathrm{T}} = 1_{vigorous}(\text{intensity})_i\left(\frac{t^9}{90^8}\right)\beta_{\text{vigorous}-\text{intensity}}. \tag{8}$$

For the secondary objective, we used model M_1. The group indicator variable categorizing the participant in either the control or athlete group was constructed using the control group as reference:

$$1_{Athlete}(\text{study group}) \coloneqq \begin{cases} 1 & \text{Former athlete} \\ 0 & \text{Control.} \end{cases} \tag{9}$$

The model M_1 part for the group indicator can be written as:

$$\mathbf{x}_i{}^{\mathrm{T}}\boldsymbol{\beta}_{\mathbf{x}} = 1_{Athlete}(\text{study group})_i\beta_{\text{study group}}. \tag{10}$$

For the supplementary analyses we utilized model M_2 for exercise volume and M_3 for intensity. The indicator variables the variables were coded as:

---

[15] We proceeded to form indicator variables according to standard coding technique used in regression modeling of categorical effects. Thus, for the first indicator variable, 1_{low}(volume), in equation (5) the value one was assigned to participants in the athlete group with total exercise volume less than 15 MET-h week. All other participants (i.e., controls and athletes with exercise volume of at least 15 MET-h/week) were given the value zero. For the second indicator variable, 1_{high}(volume), athletes with exercise volume of 15 MET-h/week or more were assigned the value one, and all other participants were given the value zero. This coding assigns the control group the value zero for both indicators, rendering it the reference group. When used in the same model the two indicator variables enable comparing low- and high-volume athletes against the reference group. For more details about dummy coding, see section 8.2.1 in Cohen et al. 2003.

$$1_{high}(\text{volume}) \coloneqq \begin{cases} 0 & \text{METh / wk} < 15 \\ 1 & \text{METh / wk} \geq 15, \end{cases} \tag{11}$$

and

$$1_{vigorous}(\text{intensity}) \coloneqq \begin{cases} 0 & \text{MET score} < 6 \\ 1 & \text{MET score} \geq 6. \end{cases} \tag{12}$$

The model parts concerning exercise volume and intensity can, thus, be written as:

$$\mathbf{x}_i{}^{\mathrm{T}}\boldsymbol{\beta}_{\mathbf{x}} = 1_{high}(\text{volume})_i\beta_{\text{high volume}} \tag{13}$$

and

$$\mathbf{x}_i{}^{\mathrm{T}}\boldsymbol{\beta}_{\mathbf{x}} = 1_{vigorous}(\text{intensity})_i\beta_{\text{vigorous}-\text{intensity}}, \tag{14}$$

$$\mathbf{w}_i(t)^{\mathrm{T}}g(t)\boldsymbol{\beta}_{\mathrm{T}} = 1_{vigorous}(\text{intensity})_i\left(\frac{t-76}{7}\right)^3\beta_{\mathrm{T}}, \tag{15}$$

respectively.

Parameter estimation was based on the method of partial maximum likelihood and conducted using the *coxph*-function of the *survival* package (3.1-12) in the R programming environment (version 4.0.2). The impact of the main covariates on fracture hazard were assessed in separate models for the three hypothesis conditions as well as exercise volume and intensity separately in for primary and tertiary hypotheses. We also used covariates to adjust the hazard ratio assessment including the time-fixed covariate of occupational class, and time-dependent covariates body height, body weight, living situation (married/cohabitating, living alone), alcohol consumption (g/month) and smoking status (current, former, never).

It is a limitation in our data that information of educational status was collected only in 2008 questionnaire study for those participants who were still alive at that time of the study corresponding to only about 20 % of the participants. Comparing the distribution of the non-missing responses to official population frequencies of educational attainment (Statistics Finland) indicated that the sample data was biased towards a higher average educational level. This suggests that the data we had available could be biased and that it would be difficult to perform adequate imputation for the data. However, we performed imputation based on the available education data, occupational class, athlete/control group status, hip fracture age and hip fracture status. The re-analyses of the primary hypothesis indicated only minor impact in the coefficients of interest. Imputation of large sections of missing data in complex data sets are generally problematic, and thus the result should be interpreted with caution. We also observed a relatively high Spearman correlation between the measures of education and occupational class[16] (r_S = 0.56) among those with intact data for these two variables, which we took to indicate that adjustment for occupational class alone was probably sufficient.

[16] Occupational classes are considered nominal variables. Hence, these classes were ordered based on expected number of years of education for the professions. The occupational class order from the lowest to highest used in calculation of the correlation was: farmer/other, unskilled workers, skilled workers, clericals and executives.

Significance testing of hazard ratios

Our goal was to investigate group differences in hazard ratios. Based on our objectives we define the following null hypotheses for significance testing.

Primary objective

- A_1: Hazard ratio for low exercise volume athletes compared to control group is equal to one.
- A_2: Hazard ratio for high exercise volume athletes compared to control group is equal to one.
- B_1: Hazard ratio for low exercise intensity athletes compared to control group is equal to one.
- B_2: Hazard ratio for vigorous exercise intensity athletes compared to control group is equal to one.

Secondary objective

- C: Hazard ratio for all athletes compared to controls is equal to one.

Tertiary objective

- D: Hazard ratio for high volume compared to low exercise volume participant groups is equal to one.
- E: Hazard ratio for vigorous exercise intensity compared to low exercise intensity participant groups is equal to one.

The alternative hypotheses were set for two-sided testing. Significance testing was based on the commonly used Wald-tests and confidence intervals for the hazard ratios were computed based on the corresponding inversions of the Wald test. We did not perform multiple testing adjustments and significance level was set at 0.05.

The confidence intervals and p-value for the hazard ratio should be interpreted as support gained by the specific hypothesis based on the current data. The confidence interval encloses values most likely under the null hypothesis. The p-value indicates whether it is likely that at least as extreme hazard ratio would be observed. As the data varies from experiment to experiment, so do the limits of the confidence intervals and the p-value. Hence, the confidence interval and p-value computed from a single study should be not interpreted as providing a definite answer, and we encourage the reader to base their conclusions about the association on more than a handful of studies.

Proportionality of hazards

Significance and detection

The validity of inference in the proportional hazards model depends on several key assumptions (for a practical overview of regression diagnostic methodology, see Therneau and Grambsch 2000). For brevity, we focus on only the proportional hazards assumption, as it provides justification for appropriate model construction.

Unbiased assessment of the hazard ratio depends on the tenability of the proportional hazards assumption (PHA). When this assumption holds, a single parameter estimate (hazard ratio) characterizes the ratio of hazard rates in two groups being compared. This brings about considerable mathematical simplification and computational convenience for modelling. For example, for the covariate used to compare athlete and control groups, it is easy to interpret the effect of a covariate, because the hazards remain equally spaced over the whole follow-up. Unfortunately, it tends to be the case that many event time processes evolve over time: e.g., the effect of vigorous-intensity exercise in the athlete group may be initially protective but later in the follow-up time turn to a hazard with respect to the fracture event. In this case the intervention effect on the hazard of the event is called time-dependent: the risk of the intervention quite literally depends on what timepoint of the follow-up is considered.

When the PHA does not hold, failure to account for change in the hazard ratio is likely to lead to biased inference if the effect does not remain constant over time. Various ways have been suggested for the

verification of the PHA (see e.g., Table 19.8. in Harrell 2001). We used the scaled Schoenfeld residual plots and tests to assess tenability of the PHA. This is a method introduced[17] by Grambsch and Therneau (1994) based on the residuals defined by Schoenfeld (1982). A plot of the residuals against follow-up time[18] (age) provides a means to assess the approximate departure from proportionality of the hazards when a spline-curve is superposed on the scatterplot. The curve can be used to locate points in time where non-proportionality is most likely to occur. A chi-square-distributed test statistic can used to assess the magnitude of departure from the null hypothesis situation of proportional hazards. It is a summary statistic that summarizes approximately the departure from proportional hazard rates. In other words, the higher the test statistic estimate, the more likely it is that hazards are not proportional over the entire follow-up period. This approach was introduced by Grambsch and Therneau (1994).

For the primary research objective, we did not find strong evidence against the proportionality assumption for exercise volume (low volume athlete vs. control $\chi^2 = 1.51$, $df = 1$, $p = 0.220$; high volume athlete vs. control $\chi^2 = 0.00$, $df = 1$, $p = 0.945$) and for low intensity athlete vs. control ($\chi^2 = 0.67$, $df = 1$, $p = 0.412$), but for vigorous-intensity athlete group indicator evidence for non-proportionality was stronger ($\chi^2 = 7.32$, $df = 1$, $p = 0.007$). The residual plot shown for the vigorous-intensity athlete group vs. control comparison in Figure MS10 indicates that initially the vigorous-intensity athlete group had a lower incidence of hip fractures relative to the control group. By age 75 years the hazards had become more similar and remained so up to age 85 years, after which the hazard of the athlete group began to rise nearly exponentially relative to the control group. In terms of the cumulative hazard the athlete group had initially a lower incidence of hip fractures to about 75 years of age, after which hazards were similar. The difference was maintained up to about 84 years of age, when the incidence in the athlete group began to rise. Thus, we decided to use the time-dependent coefficients model (M_3) in relating the hazards of the vigorous-intensity athlete to control group. The choice can be justified in terms of model fit.

For the secondary objective analysis, we assumed proportionality of hazards, as the test statistic was also not statistically significant ($\chi^2 = 2.40$, $df = 1$, $p = 0.121$). Likewise, for the unadjusted model the PHA test statistic was also non-significant ($\chi^2 = 2.68$, $df = 1$, $p = 0.102$). In studying the plots, however, the residuals suggested similar but less extreme behaviour of $\beta(t)$ at both ends of the follow-up period as we observed for the time-dependent effect for vigorous-intensity athlete vs. control group in the primary objective analysis.

For the tertiary objective, we did not find statistically significant departure from the PHA assumption in the analysis conducted for exercise volume ($\chi^2 = 1.92$, $df = 1$, $p = 0.166$). In the analysis for exercise intensity, we were in the middle ground with respect to how to interpret the results of the PHA analysis. A similar, albeit less clear pattern emerged when analysis was carried out on pooled exercise intensity data divided into vigorous and low intensity groups (see Figure MS11). Although the PHA test statistic was not statistically significant for the fully adjusted model ($\chi^2 = 3.23$, $df = 1$, $p = 0.072$), it was statistically significant for the unadjusted model ($\chi^2 = 4.97$, $df = 1$, $p = 0.026$). For this reason, we estimated time-dependent effects using model M_3 for the pooled-data analysis of exercise intensity. In Figure MS11 we can see that the highest separation between the hazard rates occurred at both ends of the follow-up time.

In summary, for the secondary objective and for the exercise volume in the primary and secondary analysis we did not find statistically significant departure from the assumption of proportionality of hazards. However,

[17] See also Therneau and Grambsch (2000) for practical examples. For slightly less technical reading and rationale, see sections 4.1.5 and 4.4.2 of Collett 2003.

[18] The Cox model is a semi-parametric model, where the prefix "semi" results from utilizing only ranking information of the follow-up time. Thus, for the assessment of proportionality it is informative to consider a modification of the exact observed follow-up time. Since the survival function is a monotonic function, the follow-up time can be replaced by the survivor function with the Kaplan-Meier transformation. The results of this transformation have been found to parallel those of the rank transformation (see Moore 2016, p. 99) particularly when there are few tied survival times.

for the exercise intensity analyses in both the secondary and tertiary analysis we observed a statistically significant departure from the proportionality of hazards assumption. A statistical explanation for this latter observation (intensity) is due to the combining of low and vigorous intensity athlete groups for the secondary objective analysis whereas they are separated for the primary and tertiary objective analyses. As the fracture rates of low intensity athletes were closer to that of the controls (see panel B of Figure in the manuscript), the difference in the hazard rates between combined low and vigorous intensity exercising athletes and controls (null hypothesis C) was smaller than between vigorous intensity athletes alone vs. the controls (null hypotheses B_2 and E).

Dealing with non-proportionality

One way to deal with non-proportional hazards is to use time-dependent effects in the regression model, as we proposed for model M_3. We next look at how to interpret such effects. Consider the binary indicator coded into variable *X*:

$$1_{vigorous}(\text{intensity}) := \begin{cases} 1 & \text{Vigorous} - \text{intensity} \\ 0 & \text{Control.} \end{cases} \quad (16)$$

For dynamically varying hazards, along with the proportional covariate effect *X* (say, β_X), we include the *X*-by-time interaction effect (β_{XT}) into the model. Now, generally at time *t* we can compute the hazard ratio as:

$$HR(t) = e^{\beta_X + t\beta_{XT}}, \quad (17)$$

and we can immediately see that *HR*(*t*) is a function of time which can take different values depending on the coefficient β_{XT}. Now, when $t = 0$, the term $t\beta_{XT} = 0$. In other words, at the start of the follow-up $HR(t) = e^{\beta_X}$, and hence we might call this main effect of *X* as 'initial level'. If the zero-point is set at some other point in time[19], we might call it the 'level' effect. On the other hand, when $t > 0$, it follows that $t\beta_{XT} \neq 0$ for non-zero β_{XT}, and as *t* increases, also $t\beta_{XT}$ can change. Because this effect is clearly dependent on time, we might call it 'time'. Note that from equation (17) the hazard can be written as: $HR(t) = e^{\beta_X + t\beta_{XT}} = e^{\beta_X} e^{t\beta_{XT}} = e^{\beta_X}\left(e^{\beta_{XT}}\right)^t$. We then report the two model effects of this product as $HR_{level} = e^{\beta_X}$ and $HR_{time} = e^{t\beta_{XT}}$. It is important to note that both HR_{level} and HR_{time} should be considered together to produce an estimate of the hazard ratio at any point of the follow-up. Neither alone is sufficient for correct interpretation of the effect.

Under the model implied by equation (17) *t* is a simple linear term, but generally changes in the effect of time may take non-linear forms. Hence, in a more general model *t* is replaced by *g*(*t*), where *g* is some function specified external to the analysis. In this case, one must construct the timepoint pointwise *HR*(*t*) using the two terms of the product and the function *g*. In case the follow-up time starts from a non-zero values (as is the case in our data) we can use the function *g*(*t* - *c*), where *c* is constant used to computationally move[20] the time origin to zero. The advantage of the time-dependent effects approach is that it permits the function to vary as a smooth function of time. Panel A in Figure MS12 shows a conceptual interpretation of the two components of the time-dependent effect. The horizontal red line indicates the level of hazard, the effect is determined at for the time point $t = 0$. The interaction effect is a curving line that first increases and then

[19] This can be achieved by adding or subtracting a constant from *t*.

[20] For example, if the minimum of observed times is at 50 units, we can use the function $g(t) = t - 50$ to place the time minimum to zero. Naturally more complex functions can be considered by e.g., taking the log of time $g(t) = \log(t - 49)$ or by considering other single variable functions of time.

decreases. It is important to specify a suitable functional for this effect to model the change appropriately over time.

To summarize the interpretation for time-dependent effect: one needs to know both the initial level (β_X) and the time-effect (β_{XT}) in order to be able to compute the hazard ratio at a specific timepoint (t) of the follow-up via $HR(T) = e^{\beta_X + g(t)\beta_{XT}}$. The initial level provides a level for the hazard ratio and the time-interaction effect (together with the index of time, t) indicate how far from this level the ratio strays at any point of the follow-up.

A significant time-interaction effect implies that the hazard rate between vigorous-intensity athlete and control groups differ from one another at some time points. As we transformed time based on an odd degree polynomial, the candidate points are at the early and late follow-up times. To find the approximate points where the hazard ratio is a significantly different from one, we computed the approximate significance of the hazard ratio at time point *t* with the Wald-statistic. The statistic is given by:

$$w = \left(\mathbf{c}^{\mathrm{T}}\boldsymbol{\beta}_{\mathrm{w}}\right)^{\mathrm{T}}\left[\mathbf{c}^{\mathrm{T}}\mathbf{V}_{w}\mathbf{c}\right]^{-1}\left(\mathbf{c}^{\mathrm{T}}\boldsymbol{\beta}_{w}\right), \qquad (18)$$

where c is a contrast vector given by the first derivatives of equation (17)[21] with respect to coefficients of interest:

$$\mathbf{c} = \frac{\partial \beta_X + g(t)\beta_{XT}}{\partial \boldsymbol{\beta}_w} = \begin{bmatrix} \frac{\partial \beta_X + g(t)\beta_{XT}}{\partial \beta_X} \\ \frac{\partial \beta_X + g(t)\beta_{XT}}{\partial \beta_{XT}} \end{bmatrix} = \begin{bmatrix} 1 \\ g(t) \end{bmatrix}, \qquad (19)$$

$\boldsymbol{\beta}_w = \begin{bmatrix} \beta_X \\ \beta_{XT} \end{bmatrix}$, and V_w is variance-covariance matrix of the parameters in $\boldsymbol{\beta}_w$. The test statistic *w* follows the chi-square distribution with one degree of freedom.

Linear approximation of a curved function

The time-transformation function for the primary objective was $g(t) = \frac{t^9}{90^8}$, which yields a line curving upwards in the interval between 85 to 90 years (solid grey line in panel B of Figure MS12). We can use the linear approximation of model-based hazards with respect to the follow-up years as:

$$\log\left[\frac{HR(t)}{HR(85)}\right] = \gamma(t - 85), \quad \text{for } t = 85, 86, \ldots, 90. \qquad (20)$$

The approximation is shown dashed dark grey line in panel B of Figure MS12. The coefficient e^{γ} can be interpreted as the approximate annual fold-increase in the HR. Other approximations are possible and can yield a more precise estimate, but we preferred this one because it has a simple interpretation involving only one parameter.

---

[21] Note that here we consider the more general situation where time is handled through the function of time *g*(*t*).

REFERENCES

Andersen PK & Gill RD. 1982. Cox's regression model for counting processes: a large sample study. Annals of Statistics: 10(4), 1100-1120.

Clayton D & Hills M. 1994. Statistical Models in Epidemiology. Oxford University Press: Oxford, UK.

Cohen J, Cohen P, West SG & Aiken LS. 2003. Applied multiple regression/correlation analysis for the behavioral sciences. 3rd Ed. Lawrence Erlbaum Associates: Mahwah, NJ.

Collett D. 2003. Modelling Survival Data in Medical Research. 2nd Ed. Chapman & Hall: Boca Raton, FL.

Cox DR. 1972. Regression Models and Life Tables (with Discussion). Journal of the Royal Statistical Society B: 34(2), 187-220.

Grambsch PA & Therneau TM. 1994. Proportional hazards tests and diagnostics based on weighted residuals. Biometrika: 81(3), 515-526.

Grolemund G & Wickham H. 2011. Dates and times made easy with lubridate. Journal of Statistical Software: 40(3): 1-25.

Harrell FE. 2001. Regression modelling strategies: With applications to linear models, logistic regression, and survival analysis. Springer: New York, NY.

Hernlund E, Svedbom A, Ivergard M, Compston J, Cooper C, Stenmark J, McCloskey EV, Jonsson B & Kanis, JA. 2013. Osteoporosis in the European Union: medical management, epidemiology and economic burden. A report prepared in collaboration with the International Osteoporosis Foundation (IOF) and the European Federation of Pharmaceutical Industry Associations (EFPIA). Archives of Osteoporosis 8(1): 136.

Kalbfleisch JD & Prentice RL. 2002. The Statistical Analysis of Failure Time Data. 2nd Ed. Wiley: Hoboken, NJ.

Klein JP & Moeschberger ML. 2003. Survival analysis: techniques for censored and truncated data. 2nd Ed. Springer: New York, NY.

Lévesque LE, Hanley JA, Kezouh A & Suissa S. 2010. Problem of immortal time bias in cohort studies: example using statins for preventing progression of diabetes. British Medical Journal 340: b5087.

Moore DF. 2016. Applied survival analysis using R. Springer: New York, NY.

R-CoreTeam. 2020. R: A language and environment for statistical computing. R foundation for statistical computing, Vienna, Austria.

Sarna S, Kaprio J, Kujala UM & Koskenvuo M. 1997. Health status of former elite athletes. The Finnish experience. Aging (Milano): 9(1-2), 35-41.

Schoenfeld D. 1982. Partial residuals for the proportional hazards regression model. Biometrika: 69(1), 239-241.

Sund R, Nurmi-Luthje I, Luthje P, Tanninen S, Narinen A & Keskimaki I. 2007. Comparing properties of audit data and routinely collected register data in case of performance assessment of hip fracture treatment in Finland. Methods of Information in Medicine: 46(5), 558-566.

Therneau TM. 2020. A package for survival analysis in R. R package version 3.1-12.

Therneau TM & Grambsch PM. 2000. Modeling Survival Data: Extending the Cox Model. Springer: New York, NY.

Zheng Z, Rahme E, Abrahamowicz & Pilote L. 2005. Survival bias associated with time-to-treatment initiation in drug effectiveness evaluation: a comparison of methods. American Journal of Epidemiology: 162(10), 1016-1023.

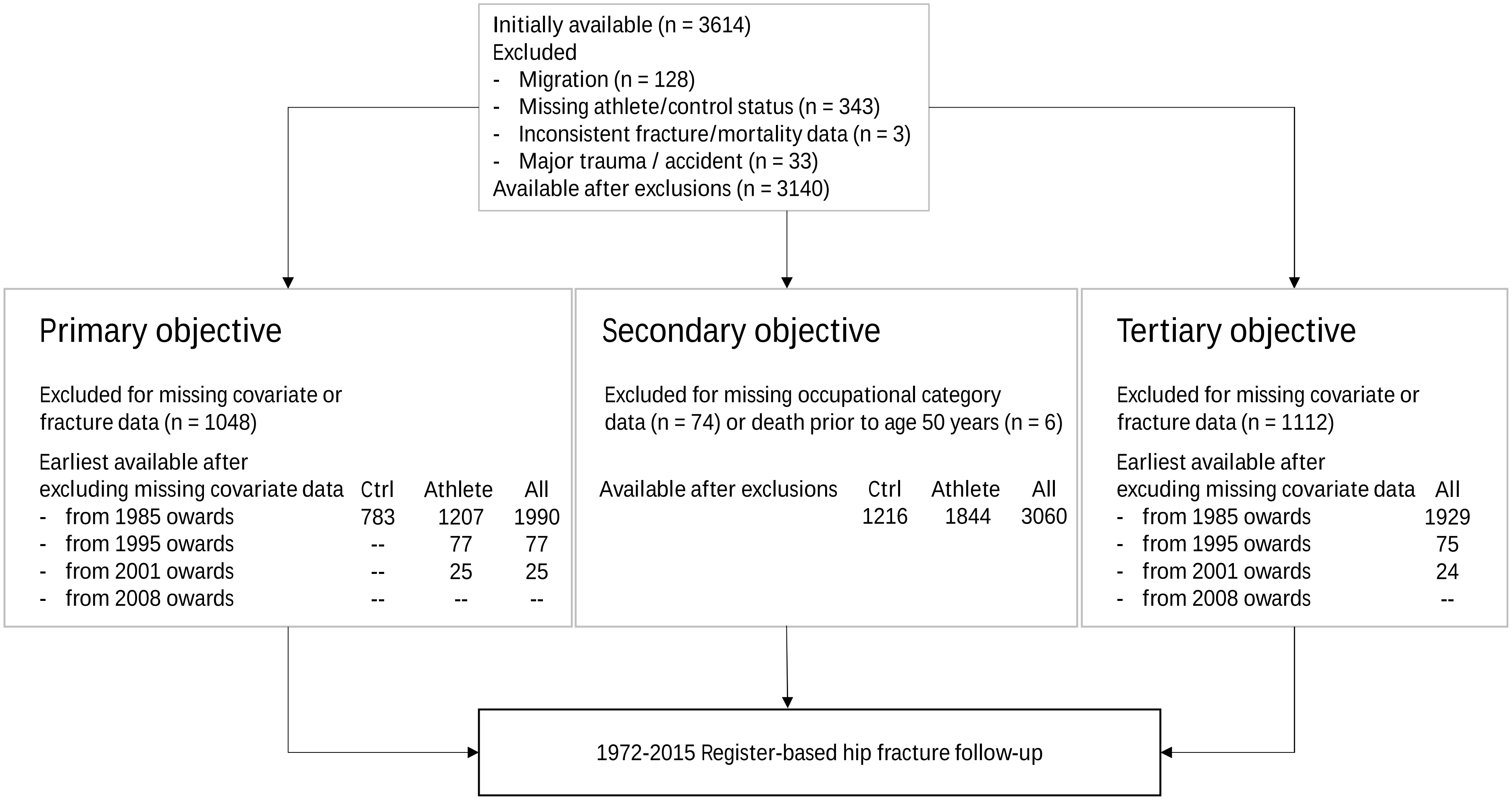

Figure MS1. Participant flow chart and sample sizes for primary, secondary, and tertiary objectives.

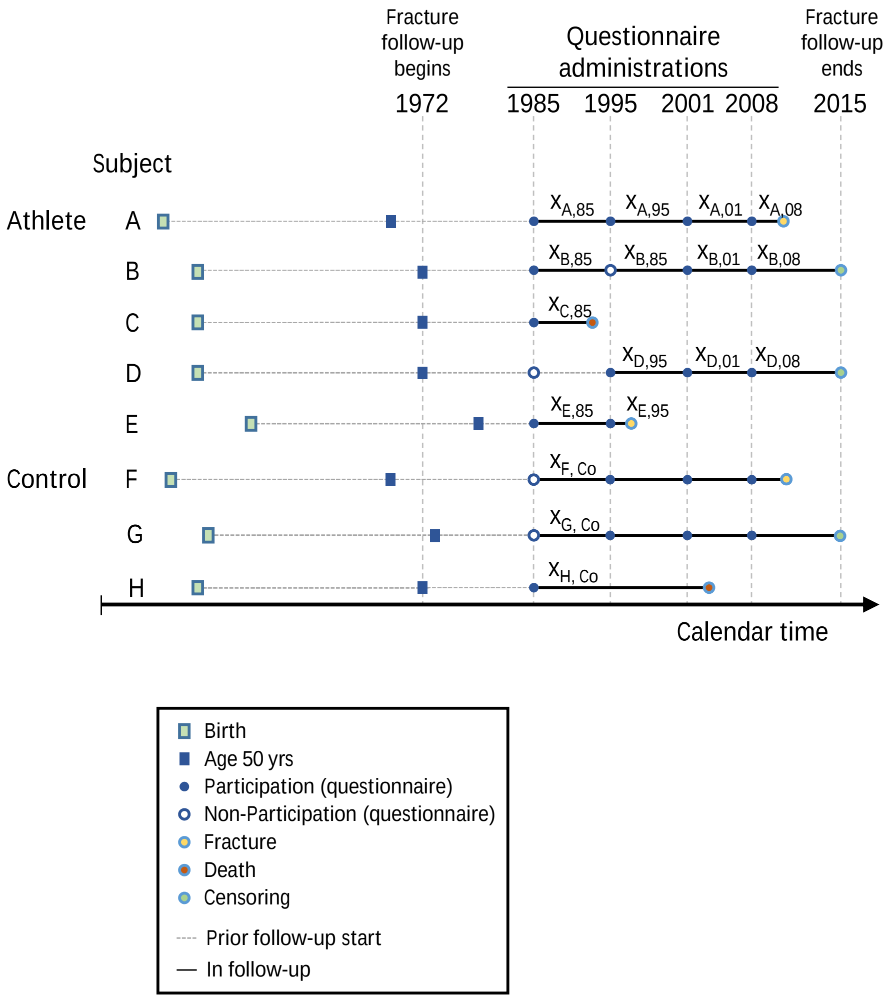

Figure MS2. Illustration of the follow-up time definition for the study of the primary hypothesis based on five theoretical cases. The quantity $x_{z,yy}$ refers to the exercise grouping *x* for participant *z* in control group (*yy* = Co) or to the measurement wave in the athlete group (yy = 1985, 1995, 2001, or 2008).

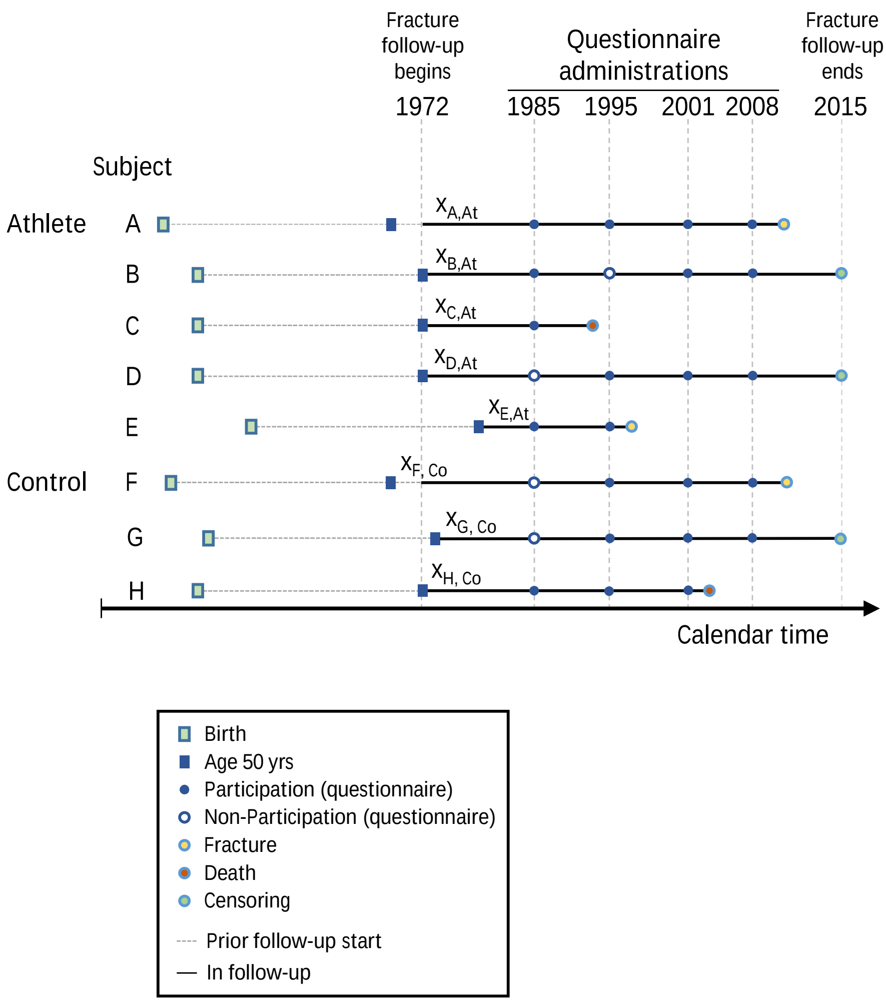

Figure MS3. Illustration of the follow-up time definition for the study of the secondary hypothesis based on five theoretical cases. The quantity $x_{z,yy}$ refers to the value of the binary indicator grouping *x* for participant *z* in the control (*yy* = Co) or athlete (yy = At) group.

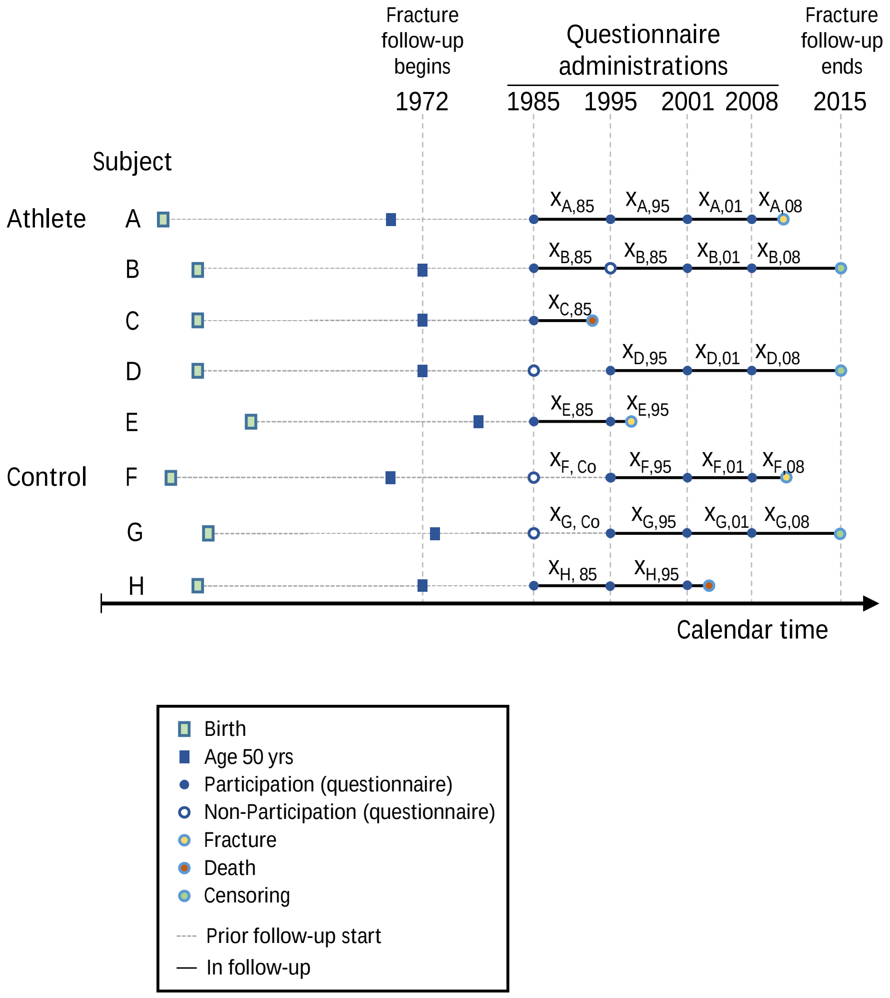

Figure MS4. Illustration of the follow-up time definition for the study of the tertiary hypothesis based on five theoretical cases. The quantity $x_{z,yy}$ refers to the exercise grouping *x* for participant *z* in measurement wave yy.

Original data format

ID	Age in follow-up		Outcome	Exercise volume			
	Entry	Exit		1985	1995	2001	2008
A	53.34	84.57	Censored=0	2	2	1	1
I	51.69	82.92	Censored=0	1	-	1	-
J	51.04	68.55	Fracture=2	1	-	2	-
K	92.95	94.17	Dead=1	-	1	-	-
L	50.36	81.12	Fracture=2	0	0	0	0
M	51.55	68.98	Dead=1	0	0	0	-

Note. ID, participant identification code.

Original data in counting process format

ID	Interval		Status	Exercise volume
	Start	End		
A	(53.34,	63.34]	0	2
A	(63.34,	69.34]	0	2
A	(69.34,	76.34]	0	1
A	(76.34,	84.57]	0	1
I	(51.69,	61.69]	0	1
I	(61.69,	67.69]	0	1
I	(67.69,	74.69]	0	1
I	(74.69,	82.92]	0	1
J	(51.04,	61.04]	0	1
J	(61.04,	67.04]	0	1
J	(67.04,	68.55]	2	2
K	(92.54,	94.17]	1	1
L	(50.36,	60.36]	0	0
L	(60.36,	66.36]	0	0
L	(66.36,	73.36]	0	0
L	(73.36,	82.12]	2	0
M	(51.55,	61.55]	0	0
M	(61.55,	67.55]	0	0
M	(67.77,	68.98]	1	0

Note. Time-intervals between consecutive measurement intervals are 10, 6 and 7 years.

Figure MS5. Illustration of the conversion of a data matrix from six fictional subjects into the counting process format.

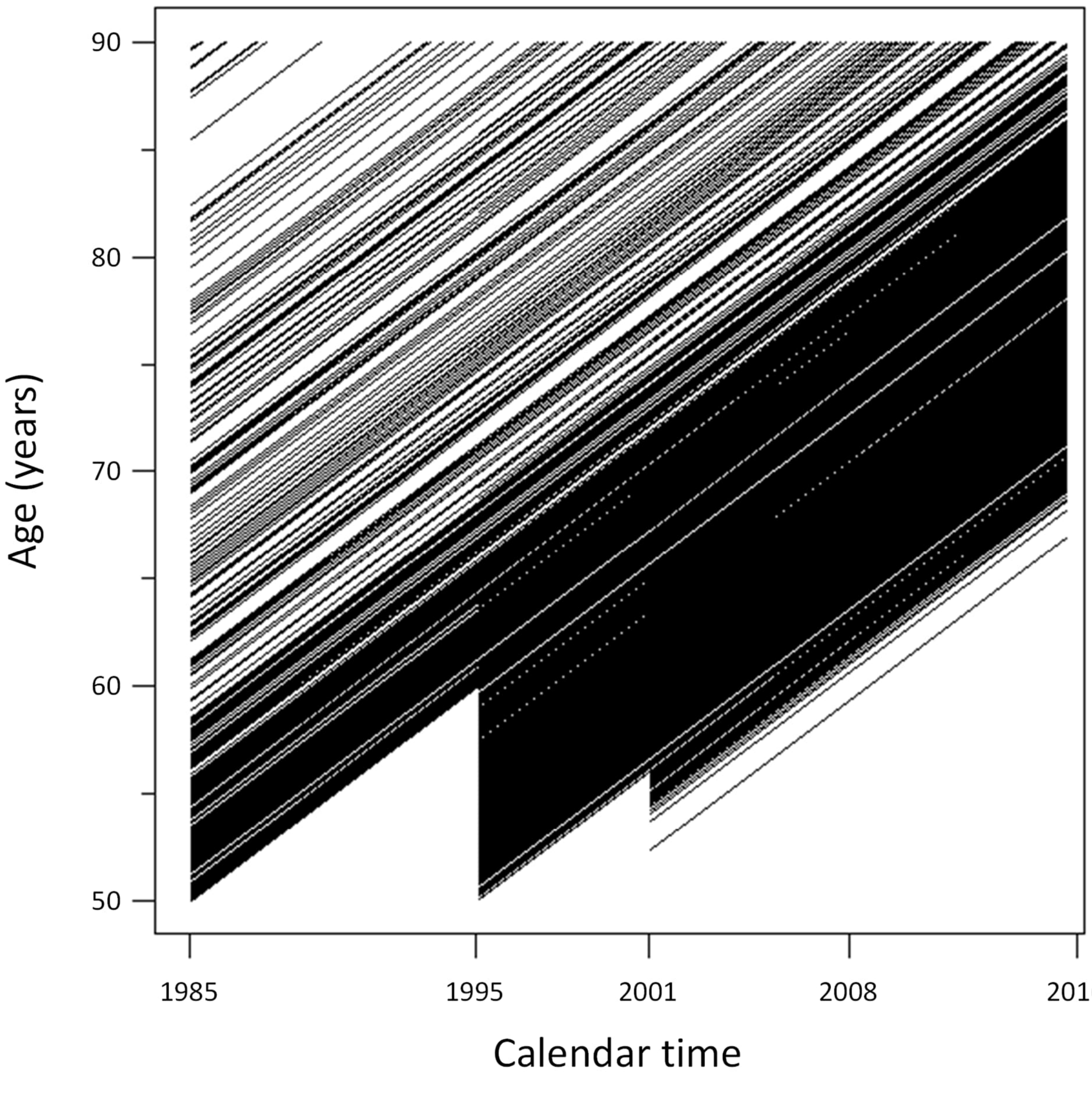

Figure MS6. Lexis diagram for participants' age according to calendar time indicating the four measurement waves and end of follow-up time for the study of the primary hypothesis. Lines indicate participation duration for *participants who were censored* at the end of follow-up time.

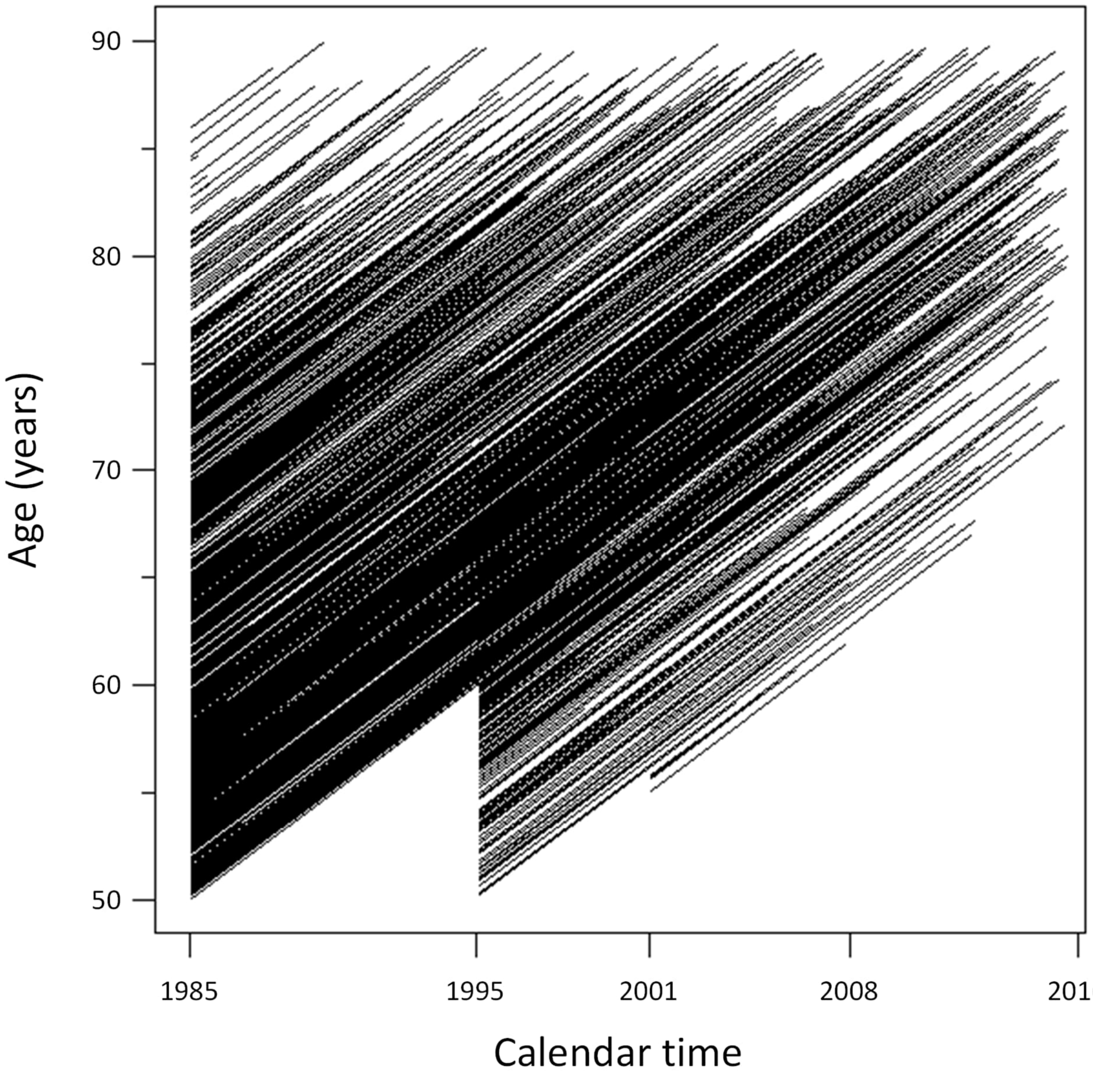

Figure MS7. Lexis diagram for participants' age according to calendar time indicating the four measurement waves and end of follow-up time for the study of the primary hypothesis. Lines indicate participation duration for *participants who died* at the end of follow-up time.

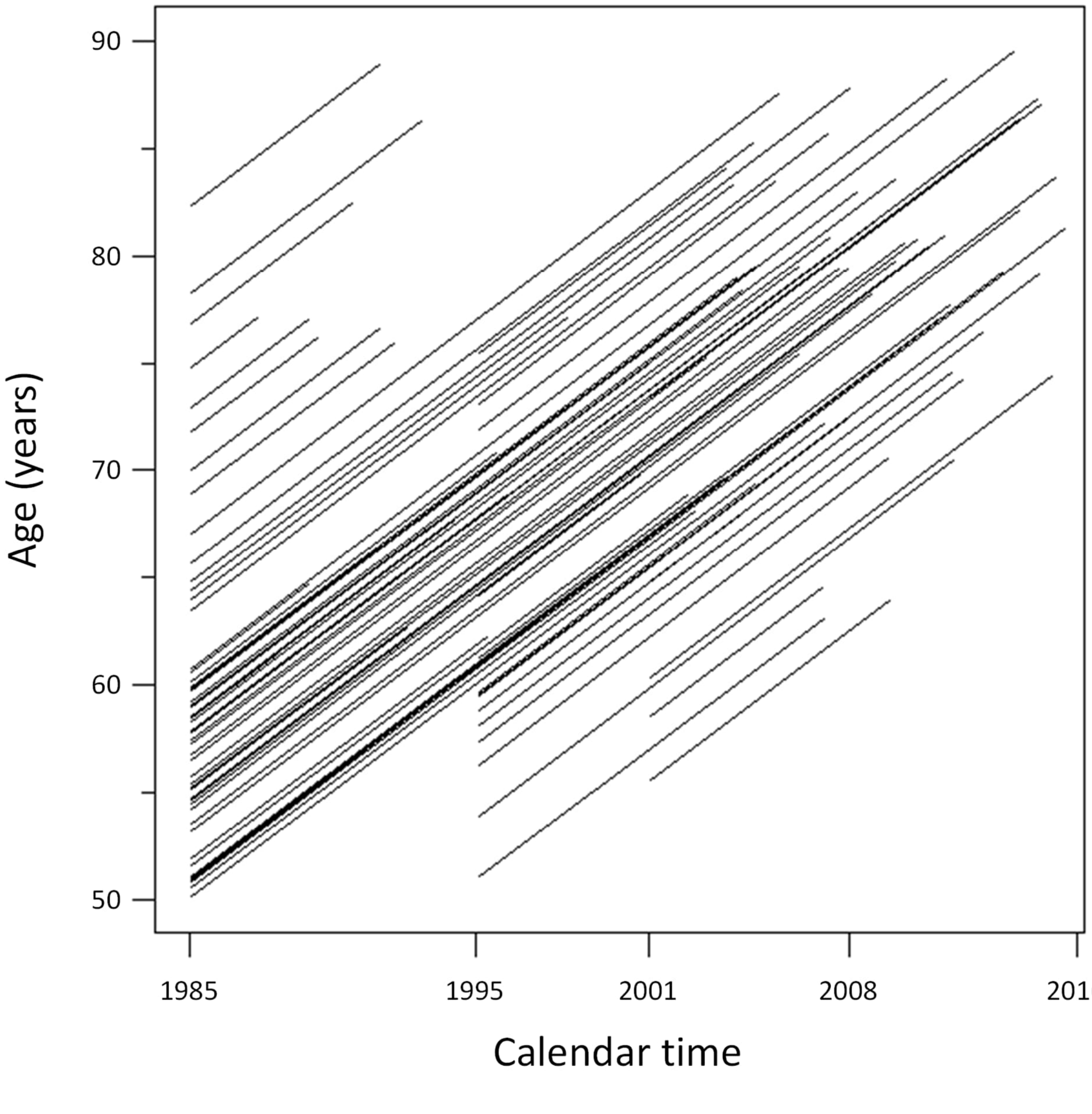

Figure MS8. Lexis diagram for participants' age according to calendar time indicating the four measurement waves and end of follow-up time for the study of the primary hypothesis. Lines indicate participation duration for *participants who sustained a fracture* at the end of follow-up time.

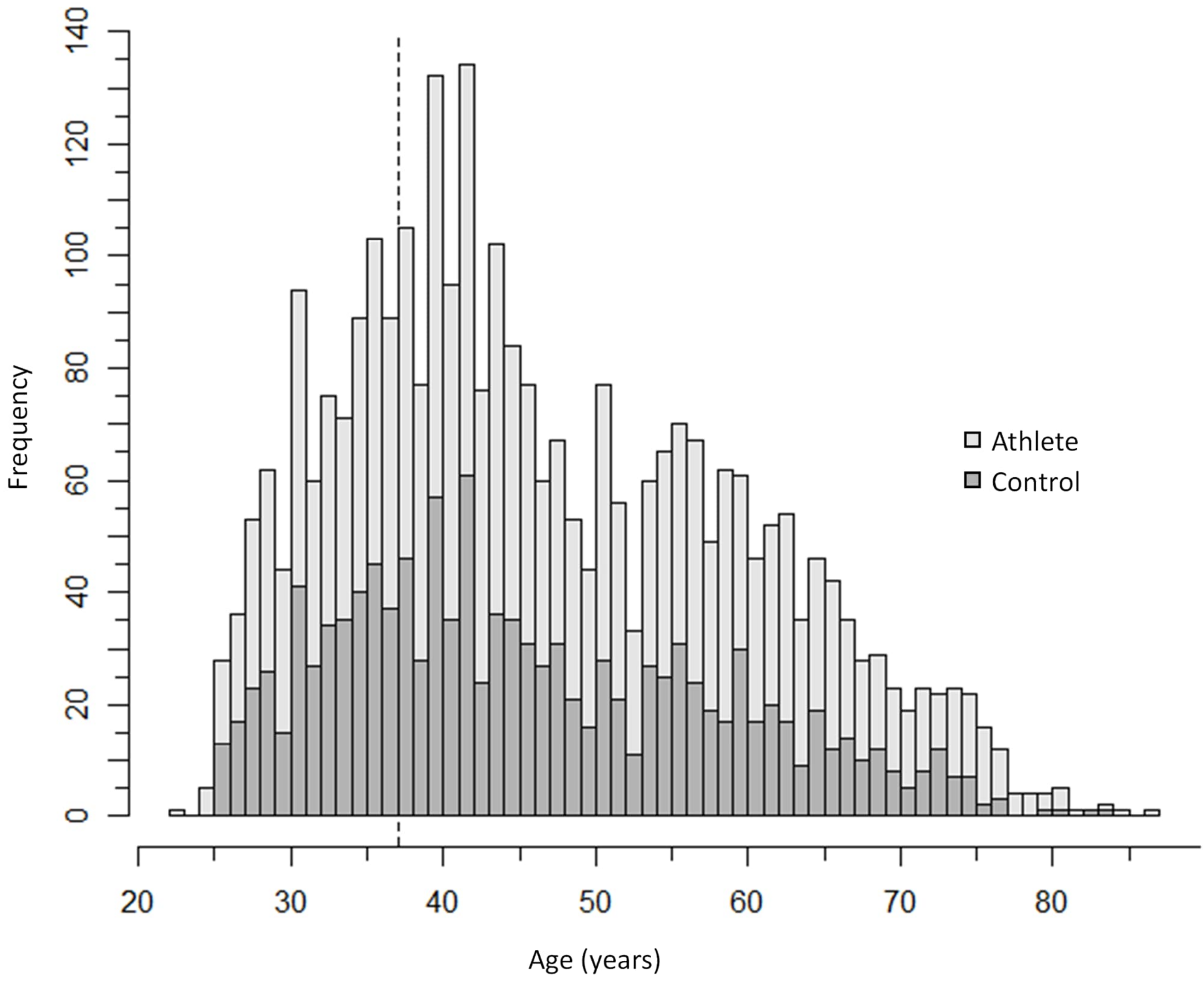

Figure MS9. Age distribution for the study participants in 1972 (N = 3060). The dashed line divides participants who would be 50 years or older in the 1985 measurement wave to the right-hand side of the figure.

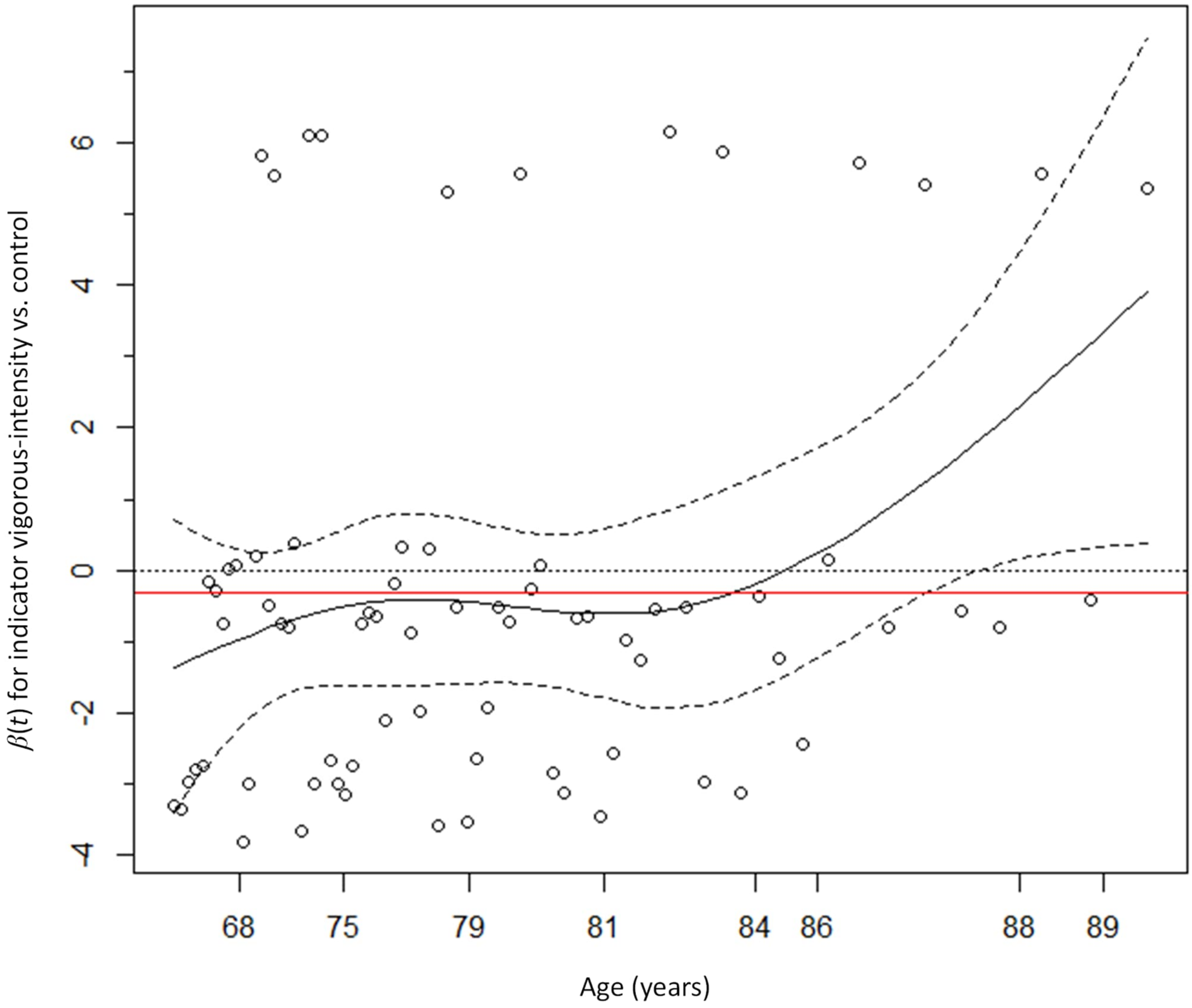

Figure MS10. Scaled Schoenfeld residuals for the indicator comparing athletes of the vigorous-intensity exercise group against the control group in the primary objective study against Kaplan-Meier transformed follow-up time (age). Solid black line is the LOESS-curve approximating the time-dependent effect based on the model residuals, dashed lines are the limits of the 95 % confidence interval for the time-dependent effect, solid red line is the time-stable effect based on the assumption of proportional hazards and the dotted line marks the line of equal hazards.

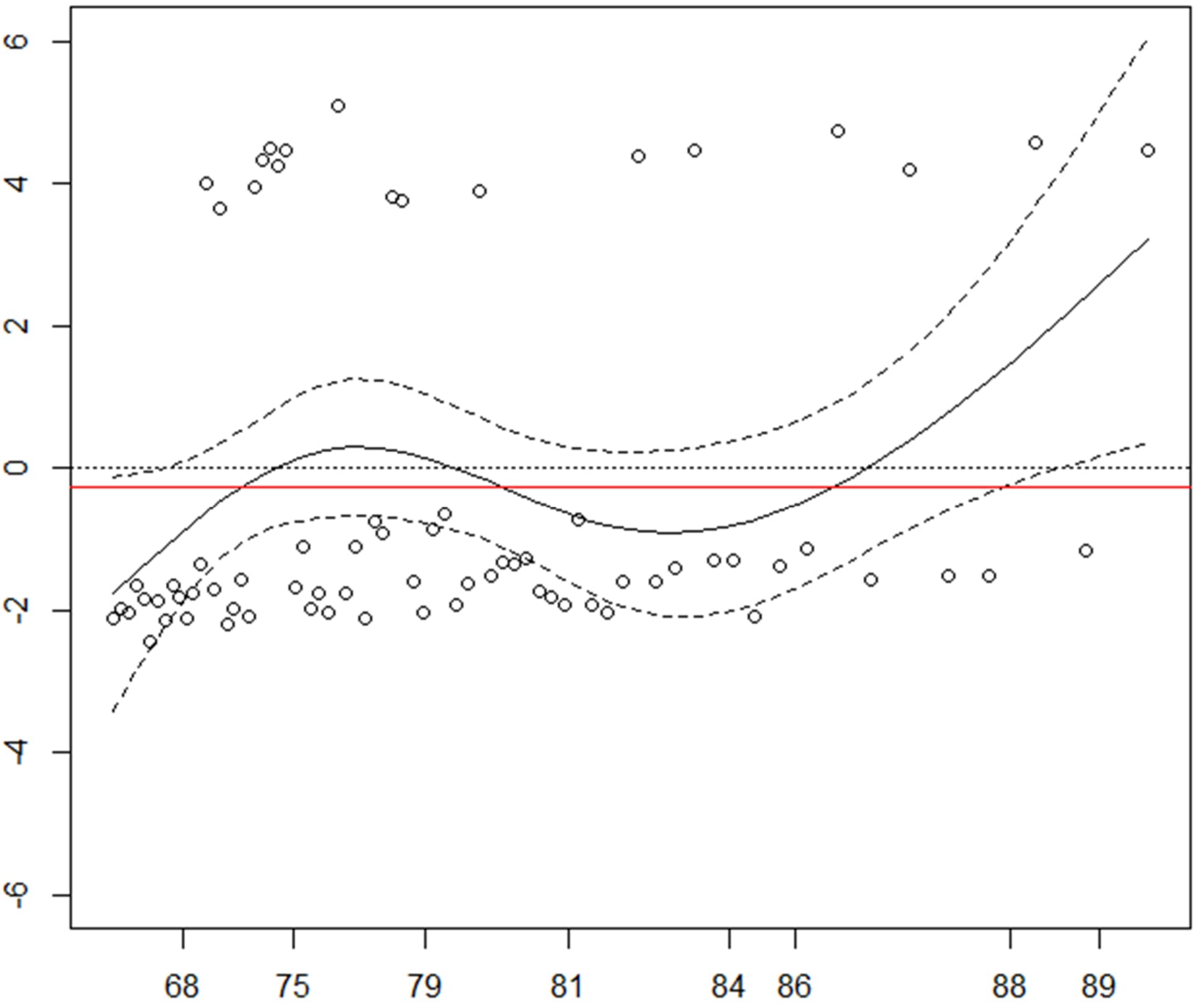

Figure MS11. Scaled Schoenfeld residuals for the indicator comparing vigorous- against low-intensity exercise groups against Kaplan-Meier transformed follow-up time (age). Solid black line is the LOESS-curve approximating the time-dependent effect, dashed lines are the limits of the 95 % confidence interval for the time-dependent effect, solid red line is the time-stable effect based on the assumption of proportional hazards and the dotted line marks the line of equal hazards.

A)

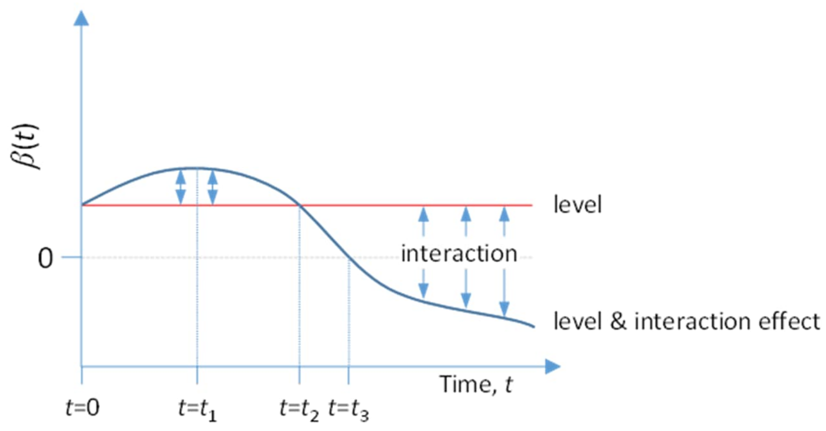

B)

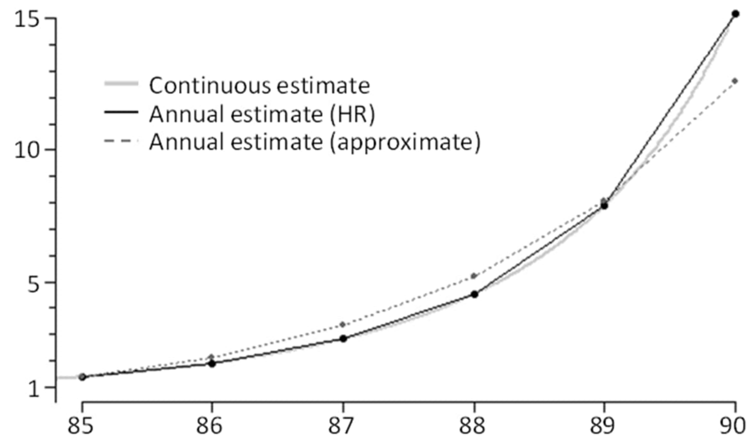

Figure MS12. *Panel A*: Interpretation of the time-dependent effect for the logarithm of an arbitrary hazard ratio, i.e., HR in the regression coefficient metric. The dotted blue line indicates level of equal hazard rates (β = 0 and HR = e^{β} = 1). The red line indicates the level effect in comparing one group (e.g., the athlete) against the reference (control). The level effect is stable over time. If the hazard rate of the athlete group varies over time relative to the control group, then the group indicator interaction with time is not zero. Thus, to obtain an estimate of the hazard ratio at a specific point in time t, both the main effect and interaction effect must be considered (black line) at t. In the figure, the total effect is equal to the main effect at time points 0 and t_2, it is larger between these time points (e.g., at t_1) and lower for $t > t_2$. The total time-dependent effect implies a higher hazard rate for the athlete group relative to controls when $t < t_3$, and a lower hazard rate when $t > t_3$. *Panel B*: Approximation of time-dependent hazard. Solid grey line indicates the continuous-time hazard estimated using $\widehat{HR}(t) = HR(\hat{\beta}_X) \times HR(\hat{\beta}_{XT})^{g(t)}$, solid black line connects dots based on annual estimates of continuous-time hazard, and dotted grey line indicates the approximation of hazard ratio. The approximation is computed as $HR(\beta_X) \times e^{\gamma t}$, where γ is the coefficient from a linear model $\log(HR_t/HR_{85}) = \gamma(t-85)$, where t is the integer-valued index of time from 85 to 90 years. The coefficient e^{γ} is a constant expressing the approximate annual fold-increase of HR over the next five years. Because of curvilinearity the HR could be more accurately modelled by adding a quadratic term in the model $\log(HR_t/HR_{85}) = \gamma_1(t-85) + \gamma_2(t-85)^2$, but the parameters γ_1 and γ_2 would no longer have a simple fold-increase interpretation.

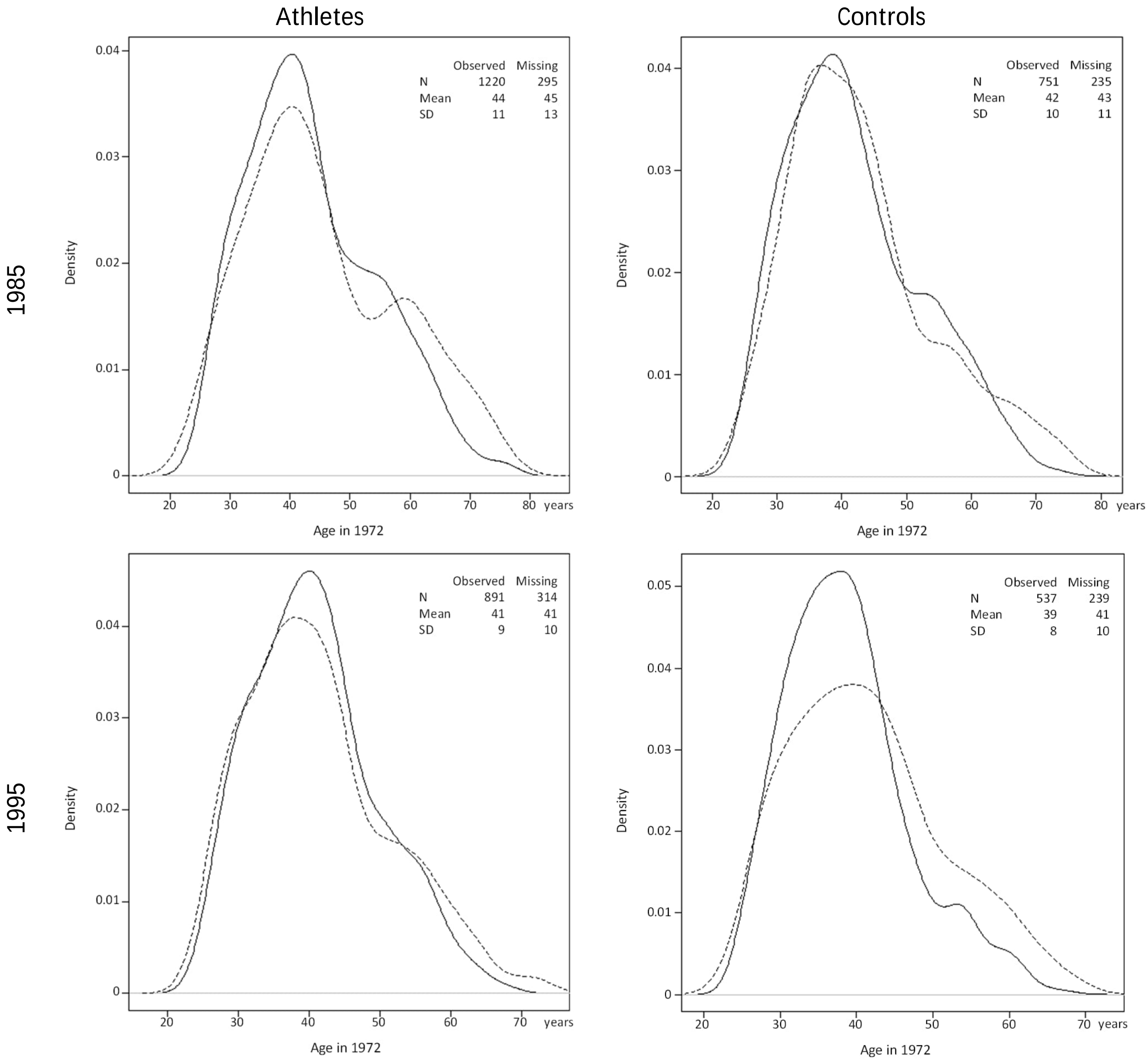

Figure MS13. Density plots of the distribution of age in 1972 for participants with observed (solid line) and missing (dashed line) MET-h/wk measurements in 1985 and 1995 among athletes and controls.

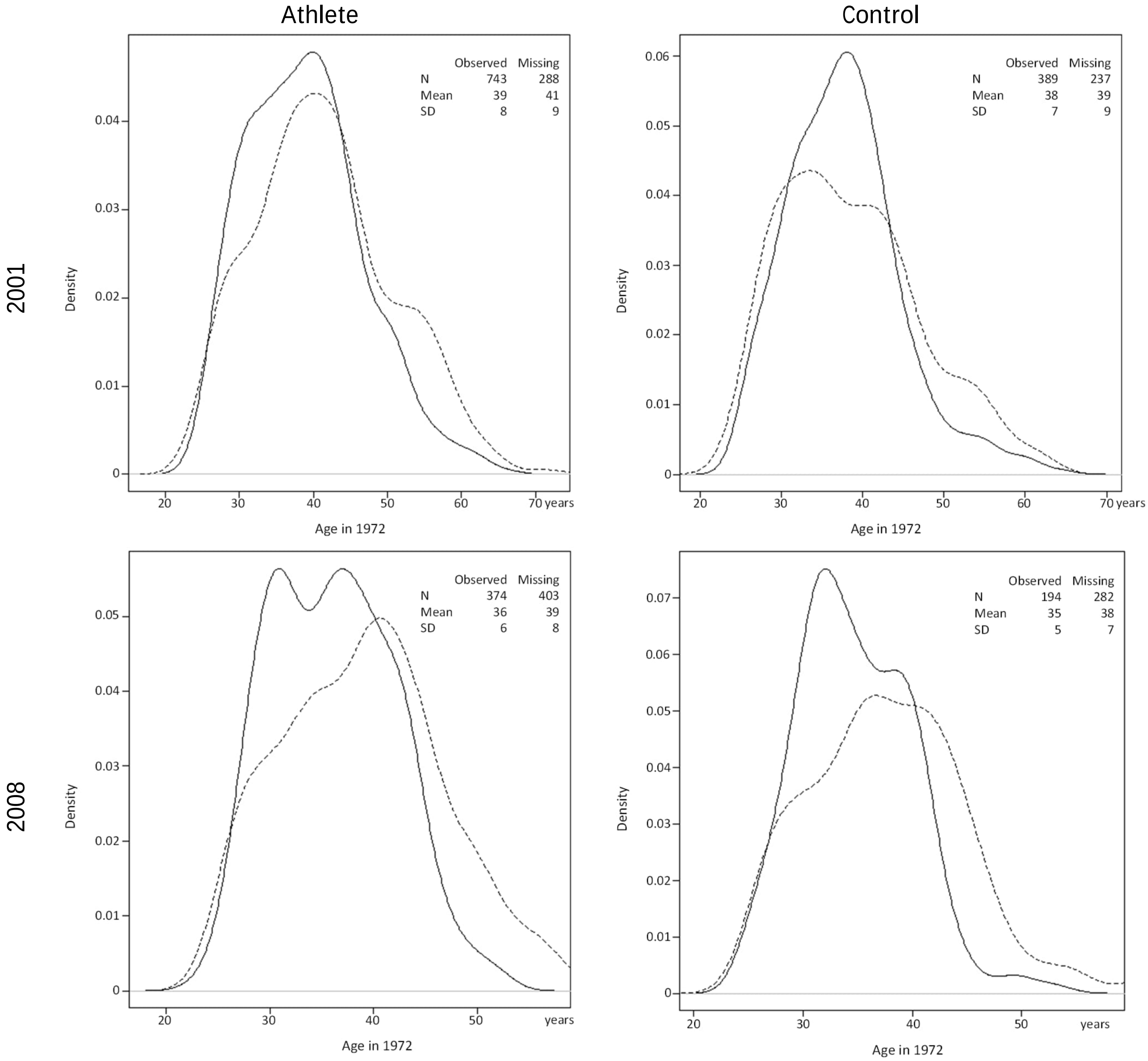

Figure MS14. Density plots of the distribution of age in 1972 for participants with observed (solid line) and missing (dashed line) MET-h/wk measurements in 2001 and 2008 among athletes and controls.

Table MS1. Frequencies for patterns of observed exercise data measured in four questionnaire waves (1985, 1995, 2001, 2008) according to follow-up endpoints (fracture, death, or censoring).

Response pattern		Interval for observed endpoint			
		1985 - 1995	1995 - 2001	2001 - 2008	2008 →
Observed	Replacement	f / d	f / d	f / d	f / d / c
× - - -	× × × ×	8 / 186	1 / 69	2 / 35	-- / 18 / 41
× × - -	× × × ×	2 / --	1 / 58	1 / 42	1 / 12 / 27
× × × -	× × × ×	-- / --	-- / --	7 / 74	9 / 58 / 139
× × × ×	× × × ×	2 / 117	3 / 108	12 / 110	15 / 141 / 557
- × × ×	- × × ×	-- / --	-- / --	-- / --	1 / 1 / 13
- - × ×	- - × ×	-- / --	-- / --	-- / --	-- / 3/ 6
- × - ×	- × × ×	-- / --	-- / --	-- / --	-- / 1 / 2
- × × -	- × × ×	-- / --	-- / --	2 / 7	1 / 5 / 14
- - × -	- - × ×	-- / --	-- / --	1 / 7	-- / 1 / 7
- × - -	- × × ×	-- / --	1 / 5	-- / 11	-- / 5 / 8
× - × ×	× × × ×	-- / --	-- / --	-- / --	-- / 8/ 28
× - - ×	× × × ×	-- / --	-- / --	1 / --	-- / -- / 13
× - × -	× × × ×	-- / --	-- / --	1 / 17	1 / 17 / 30
× × - ×	× × × ×	-- / --	-- / --	-- / --	-- / 5 / 13

Note. Under observation pattern: cross = observed, single dash = missing. The order of symbols from first to last refers to the 1985, 1995, 2001 and 2008 measurement waves, respectively. Frequencies shown for endpoints f = facture, d = death, and c = censored. Null frequency is indicated by double dashing.

Table MS2. Number and percentage of surviving participants without fracture (eligible) in the athlete and control groups responding to exercise items of the questionnaire in each measurement wave.

	Athlete			Control		
Year	Observed	%	Eligible	Observed	%	Eligible
Intensity (METs)						
1985	1234	81	1515	984	100	984
1995	812	67	1205	776	100	776
2001	743	72	1031	626	100	626
2008	374	48	777	476	100	476
Intensity, pooled						
1985	1196	79	1515	720	73	984
1995	812	67	1205	437	56	776
2001	724	70	1031	391	62	626
2008	374	48	777	196	41	476
Volume (MET-h/wk)						
1985	1220	81	1515	751	76	984
1995	891	74	1205	537	69	776
2001	743	72	1031	389	62	626
2008	374	48	777	194	41	476

Table MS3. Frequencies of participants in occupational classes among those with observed and missing MET-h/wk in the questionnaire studies (1985-2008).

Occupational Class	Athletes					Controls				
	1972[a]	1985	1995	2001	2008	1972[a]	1985	1995	2001	2008
Observed METh/wk										
Executives	459 (25)	297 (25)	239 (27)	214 (29)	115 (31)	114 (9)	86 (11)	67 (12)	54 (14)	32 (16)
Clericals	710 (39)	505 (42)	354 (40)	299 (40)	152 (41)	272 (22)	198 (26)	147 (27)	114 (29)	60 (31)
Skilled	543 (29)	346 (29)	243 (27)	186 (25)	86 (23)	482 (40)	313 (42)	228 (42)	157 (40)	75 (39)
Unskilled	41 (2)	24 (2)	16 (2)	13 (2)	6 (2)	116 (10)	39 (5)	25 (5)	13 (3)	5 (3)
Farmers	88 (5)	28 (2)	37 (4)	29 (4)	14 (4)	227 (19)	115 (15)	70 (13)	51 (13)	22 (11)
Other	3 (<1)	0 (0)	2 (<1)	2 (<1)	1 (<1)	5 (<1)	0 (0)	0 (0)	0 (0)	0 (0)
Missing METh/wk										
Executives	--	76 (26)	74 (24)	58 (20)	91 (20)	--	13 (6)	18 (8)	21 (9)	30 (11)
Clericals	--	106 (36)	129 (41)	116 (40)	165 (40)	--	34 (14)	42 (18)	49 (21)	64 (23)
Skilled	--	84 (28)	88 (28)	95 (33)	122 (33)	--	99 (42)	104 (44)	101 (43)	126 (45)
Unskilled	--	7 (2)	7 (2)	7 (2)	9 (2)	--	39 (17)	31 (13)	32 (14)	22 (8)
Farmers	--	19 (6)	15 (5)	11 (4)	15 (4)	--	48 (20)	44 (18)	34 (14)	40 (14)
Other	--	3 (1)	1 (<1)	1 (<1)	1 (<1)	--	2 (1)	0 (0)	0 (0)	0 (0)

[a]Occupational data was available for all participants (from Central Population Registry of Finland).
