# Supplementary material for: Longitudinal Associations of High‐Volume and Vigorous‐Intensity Exercise With Hip Fracture Risk in Men
Source: J Bone Miner Res. 2022 Jul 6;37(8):1562–70. doi: 10.1002/jbmr.4624 (PMC9544739; doi:10.1002/jbmr.4624)
Supplement: Supplementary file 2 — Fig. S1. Total exercise volume (A) and average exercise intensity (B) by group, and proportion of athletes and controls reaching cut points of high total exercise volume of ≥15 MET‐hour/week (C) and vigorous average exercise intensity of ≥6 METs at least 75 minutes/week (D) at different time points. Values within bars are means (A, B) and mean percentages (C, D) with 95% confidence intervals in parentheses. p < 0.001 in all pairwise comparisons between groups (A–D). Total exercise volume (A: cf. Kontro et al. Eur J Sport Sci, DOI: 10.1080/17461391.2020.1761889). [file JBMR-37-1562-s002.pdf]

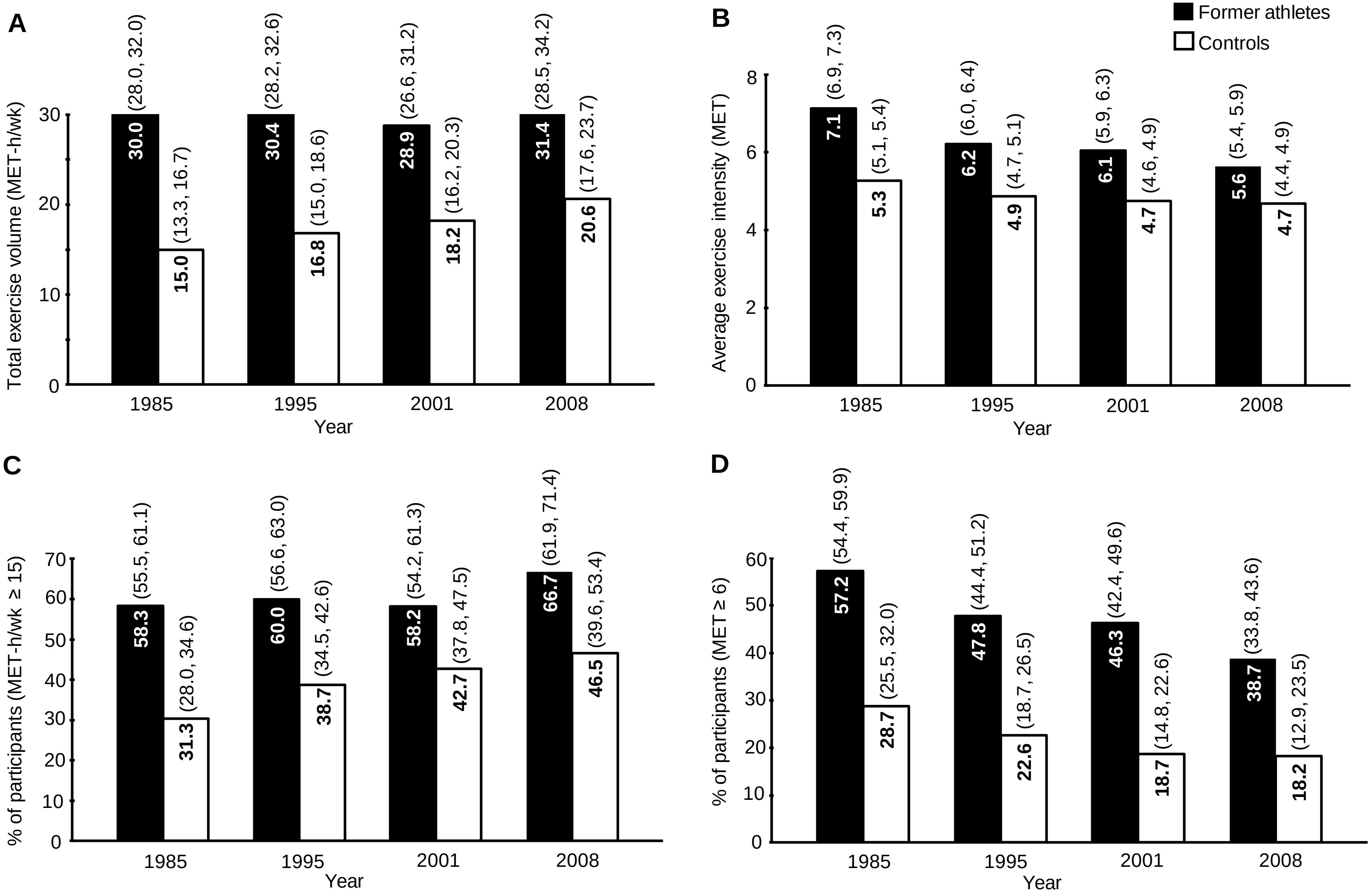

Supplemental Figure 1. Total exercise volume (A) and average exercise intensity (B) by group, and proportion of athletes and controls reaching cut points of high total exercise volume of ≥ 15 MET-h/wk (C) and vigorous average exercise intensity of ≥ 6 METs at least 75min/wk (D) at different time points. Values within bars are means (A, B) and mean percentages (C, D) with 95% confidence intervals in parentheses. $p < 0.001$ in all pairwise comparisons between groups (A-D). Total exercise volume (Panel A, cf. Kontro et al. Eur J Sport Sci, DOI: 10.1080/17461391.2020.1761889).
