# Supplementary material for: Longitudinal Associations of High‐Volume and Vigorous‐Intensity Exercise With Hip Fracture Risk in Men
Source: J Bone Miner Res. 2022 Jul 6;37(8):1562–70. doi: 10.1002/jbmr.4624 (PMC9544739; doi:10.1002/jbmr.4624)
Supplement: Supplementary file 3 — Fig. S2. Nelson‐Aalen cumulative fracture hazard curves of hip fractures for the pooled sample (athletes plus controls) stratified into two groups based on total exercise volume (A) and average exercise intensity (Panel B). Hazard ratios (HRs) and their 95% confidence intervals (CIs) are shown in the inset tables. The models were controlled for occupation, height, weight, living situation, alcohol use and smoking history. Nelson‐Aalen curves of hip fracture risks were adjusted by possible changes in exercise level during follow‐up. Time‐dependent effect observed for exercise intensity (B) is partitioned into two components: initial HR is the hazard ratio at the start of the follow‐up period and time HR refers to risk modification over time. Hip fractures were followed starting from the first questionnaire participation until time of death, diagnosis of hip fracture or end of follow‐up on December 31, 2015. Exercise and other characteristics were obtained from questionnaire studies in 1985, 1995, 2001 and 2008. See Table 1 footnote for description of calculation of exercise volume and intensity with MET (metabolic equivalent) values. [file JBMR-37-1562-s003.pdf]

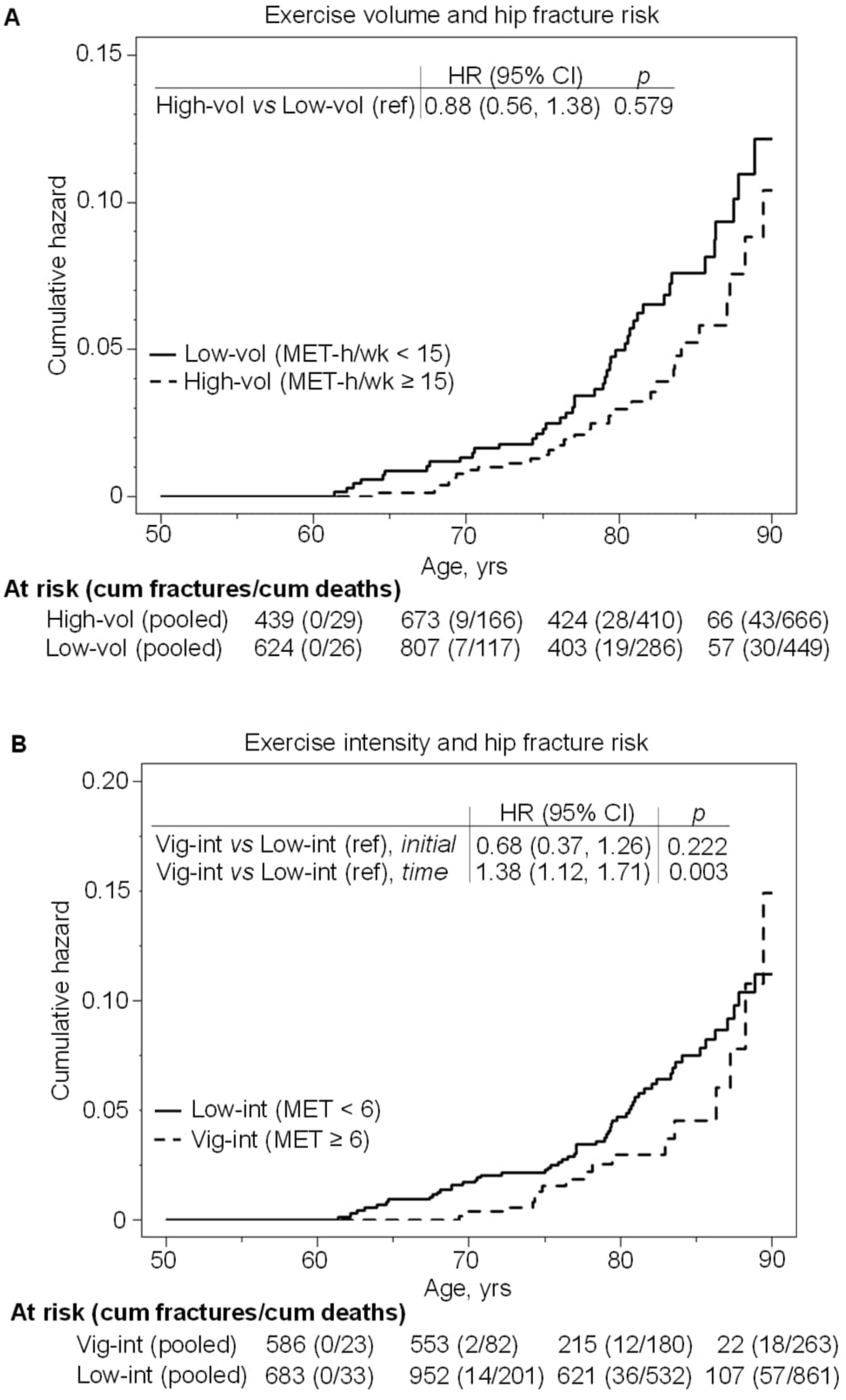

Supplemental Figure 2. Nelson-Aalen cumulative fracture hazard curves of hip fractures for the pooled sample (athletes plus controls) stratified into two groups based on total exercise volume (Panel A) and average exercise intensity (Panel B). Hazard ratios (HR) and their 95% confidence intervals (CI) are shown in the inset tables. The models were controlled for occupation, height, weight, living situation, alcohol use and smoking history. Nelson-Aalen curves of hip fracture risks were adjusted by possible changes in exercise level during follow-up. Time-dependent effect observed for exercise intensity (Panel B) is partitioned into two components: initial HR is the hazard ratio at the start of the follow-up period and time HR refers to risk modification over time. Hip fractures were followed starting from the first questionnaire participation until time of death, diagnosis of hip fracture or end of follow-up on 31 December, 2015. Exercise and other characteristics were obtained from questionnaire studies in 1985, 1995, 2001 and 2008. See Table 1 footnote for description of calculation of exercise volume and intensity with MET (metabolic equivalent) values.
